# Nano‐enabled Tumor Systematic Energy Exhaustion via Zinc (II) Interference Mediated Glycolysis Inhibition and Specific GLUT1 Depletion

**DOI:** 10.1002/advs.202103534

**Published:** 2021-12-16

**Authors:** Sixuan Wu, Kaixiang Zhang, Yan Liang, Yongbin Wei, Jingyi An, Yifei Wang, Jiali Yang, Hongling Zhang, Zhenzhong Zhang, Junjie Liu, Jinjin Shi

**Affiliations:** ^1^ School of Pharmaceutical Sciences Zhengzhou University Zhengzhou 450001 P. R. China; ^2^ Key Laboratory of Targeting Therapy and Diagnosis for Critical Diseases Zhengzhou 450001 P. R. China; ^3^ Key Laboratory of Advanced Drug Preparation Technologies Ministry of Education Zhengzhou 450001 P. R. China; ^4^ State Key Laboratory of Esophageal Cancer Prevention & Treatment Zhengzhou 450001 P. R. China

**Keywords:** GLUT1 depletion, glycolysis inhibition, starvation therapy, systematic energy exhaustion, zinc (II) interference

## Abstract

Despite the promise of tumor starvation therapies, they are often associated with nonspecific and incomplete energy blockade. Here, a novel paradigm of starvation therapy is proposed to synergize the “Zn^2+^ interference”‐mediated glycolysis inhibition and Zn^2+^‐activating GLUT1 (Glucose transporter 1) tumor specific depletion for systematic energy exhaustion. It is discovered that ZIF‐8 (zinc imidazolate metal–organic frameworks ) can induce abrupt intracellular Zn^2+^ elevation preferentially in melanoma cells, and then achieve effective glycolysis blockade through “Zn^2+^ interference”‐triggered decrease of NAD^+^ and inactivation of GAPDH, making it a powerful tumor energy nanoinhibitor. Meanwhile, Zn^2+^‐activating DNAzymes for specifically cleaving GLUT1 mRNA is designed. This DNAzyme can only be activated under intracellular Zn^2+^ overloading, and then directionally cut off glucose supply, which further restrains the adaptive up‐regulation of glycolytic flux after glycolysis inhibition in tumors. Afterward, DNAzymes are loaded in ZIF‐8 concurrently tethered by hyaluronic acid (HA), constructing a “nanoenabled energy interrupter ”. Such a rational design presents a preferential accumulation tendency to tumor sites due to the active CD44‐targeting mechanisms, specifically achieves remarkable systematic energy exhaustion in melanoma cells, and affords 80.8% in tumor growth suppression without systemic toxicity in vivo. This work verifies a fascinating therapeutic platform enabling ion interference‐inductive starvation strategy for effective tumor therapy.

## Graphical abstract

A “nano‐enabled energy interrupter” with capability of specific Zn(II) homeostasis disrupting in malignant melanoma is designed. The specific Zn(II) overloading in tumor cells can inhibit glycolysis and initiate GLUT1 depletion for energy exhaustion induced tumor therapy, while the “nanoenabled energy interrupter” has negligible impact on Zn(II) homeostasis and energy metabolism in normal cells.

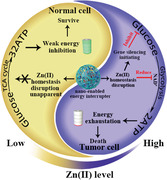

## 1 Introduction

Starvation therapy has aroused considerable interest as a burgeoning modality for the clinical management of tumors in recent years.^[^ ^1^ ^]^ It is well‐known that starvation treatment can inhibit tumors via ingredient consumption (i.e., glucose exhaustion, oxygen scavenging, and nutrition intake blockade) or blood vessel occlusion that are all essential for tumor growth.^[^ ^2^ ^]^ Among these, directly cutting off blood supply at the tumor sites via two typical protocols, i.e., intratumoral vascular damage or intravascular aggregate‐mediated embolism, is of much more concern to the starvation therapy as blood vessels are essential for the tumor to gain energy.^[^ ^3^ ^]^ However, these strategies for blood vessel occlusion also confront several intractable drawbacks, including vascular obstruction in normal tissue, lower efficacy of vascular occlusion triggered by rapid blood flow in tumor tissue.^[^ ^3^ ^]^ Another is to reduce glucose uptake by tumor cells. As a typical glucose depletory, glucose oxidase (GOx) has been vigorously studied for tumor starvation therapy.^[^ ^4^ ^]^ Nevertheless, the complexity of tumor microenvironment, such as hypoxia, high protease level, severely limits the treatment efficiency of GOx‐based starvation therapy.^[^ ^5^ ^]^ Moreover, persistent and nonspecific glucose exhaustion always leads to unexpected systemic hypoglycemia,^[^ ^6^ ^]^ which also impeded the clinical translation of GOx‐based therapeutics. Fortunately, the in‐depth insight into starvation therapy encourages us to pioneer a novel alternative strategy for energy deprivation of tumor cells.

Biological metal ions (e.g., Ca^2+^, Zn^2+^, Cu^2+^) playing well‐established biochemical and nutritional roles are essential for cell growth.^[^ ^7^ ^]^ While their abnormal accumulation in cells may result in an irreversibly killing effect.^[^ ^8^ ^]^ Therefore, intracellular ion overloading, for example, Ca^2+^ overloading, has been exploited for combating multiple types of tumors.^[^ ^9^ ^]^ Compared with Ca^2+^, Zn^2+^ as the most prevalent coenzyme factor, plays a more basic role in cell growth via manipulating energy metabolism, gene expression, and genomic stability, et al.^[^ ^10^ ^]^ However, Zn^2+^ overloading‐mediated tumor therapy and the underlying therapeutical mechanism have rarely been reported in depth. Recently, studies showed Zn^2+^ could act as an energy predator in neurons via irreversibly hinder energy production process, specifically, attributable to NAD^+^ loss and inhibition of glycolysis.^[^ ^11^ ^]^ Inspired by this, we deem that “Zn^2+^ interference” strategy can be developed for an alternative tumor starvation therapy, simultaneously overcoming the above drawback of starvation therapy. More meaningful, due to the higher energy craving and the lower tolerance threshold of Zn^2+^ fluctuation, tumor cells are expected to more susceptible to Zn^2+^ accumulation than healthy cells,^[^ ^12^ ^]^ guaranteeing a more efficient and safer performance of energy deprivation compared to other starving strategies.

Given the potential energy interference effect of Zn^2+^ on tumor cells, we propose that zinc‐containing nanoparticles may serve as attractive candidate materials for developing a tumor starvation platform. Nanoscaled Zeolitic imidazolate framework‐8 (ZIF‐8), composed of Zn^2+^ and dimethylimidazole, has been widely developed for drug delivery.^[^ ^13^ ^]^ Benefiting from the pH‐triggered structural collapse, ZIF‐8 can concurrently release Zn^2+^ and therapeutic drugs into the cytoplasm under acidic lysosomal stimulation.^[^ ^13a^ ^]^ Such a feature endows ZIF‐8 typical intracellular Zn^2+^ interference ability that is rarely investigated, deserving to be explored and utilized in‐depth. Worth thinking about further, the blockade of intracellular glycolytic pathway often leads to the adaptive upregulation of the glycolytic flux and then results in the upregulation of glucose transporters 1 (GLUT1) expression in tumors,^[^ ^14^ ^]^ which needs further GLUT1 blockade. While the general application of GLUT1 inhibitors including diclofenac (DC) and siRNA often brings systemic energy metabolism disorders.^[^ ^15^ ^]^ Therefore, it is also highly desired to locally suppress the expression of GLUT1 on tumor cells. To achieve local GLUT1 suppression, we designed a highly efficient Zn^2+^‐activating GLUT1 mRNA‐cleaving DNAzyme (defined as GD), which only can be specifically activated by our “Zn^2+^ interference” strategy for inhibiting GLUT1 expression in tumor cells. Comparing with traditional siRNA‐based gene silencing strategy, DNAzymes are catalytic nucleic acids that can mimic the function of endonucleases for cleavage of specific mRNA with multiple turnovers, which may be more effective for GLUT1 gene silencing on a per molecule basis.^[^ ^13^, ^16^ ^]^ Interestingly, by integrating DNAzymes with our nanoenable Zn^2+^ interference strategy, the inherent defects of DNAzymes with ion as coenzyme factor when applied in vivo, including insufficient cofactor supply and poor intracellular delivery efficacy,^[^ ^13a^ ^]^ can be cleverly solved.

**Scheme 1:**
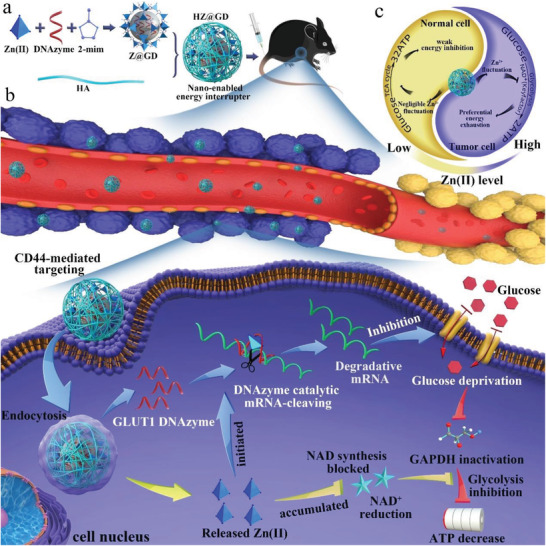
The scheme of dual gate‐controlled “nano‐enabled energy interrupter” with Zn^2+^ interference‐mediated glycolysis inhibition and Zn^2+^‐activating GLUT1 depletion for tumor systematic energy exhaustion. a) a schematic illustration of the procedure for preparing Z@GD nanoparticles using zinc sources, dimethylimidazole, DNAzymes, then modifying the Z@GD nanoparticles with hyaluronic acid (HA) to form HZ@GD nanoparticles. b) Owing to the enhanced permeability and retention (EPR) effect and CD44‐mediated actively targeting mechanism of tumor, HZ@GD nanoparticles can preferentially accumulate in tumor but not normal tissues to minimize systemic toxicity of nanoparticles. After arriving in tumor, the HZ@GD nanoparticles can be taken up by tumor cells in CD44‐mediated endocytosis manner. Benefiting from the degradation of HAase and acidic pH, Zn^2+^, and DNAzymes can be simultaneously released into the cytoplasm. On the one hand, intracellular Zn^2+^ overloading can efficiently inhibit glycolysis pathway. On the other hand, adequate amounts of Zn^2+^ can specifically activate DNAzymes to cleave GLUT1 mRNA for cutting off glucose supply. Finally, a novel “top‐down” strategy of energy blockade achieves specific and complete energy exhaustion of tumor cells. c) A tai chi schematic is used to generalize that HZ@GD nanoparticles can result in selective Zn^2+^ overloading in tumor cells, ultimately achieving preferential energy blockade in tumor cells.

Herein, a dual gate‐controlled “nanoenabled energy interrupter” that contains a zinc core (ZIF‐8), a designed nucleic acid drug (GD), and a tumor‐targeting hydrophilic shell (Hyaluronic acid, HA) was rationally constructed for systematic energy exhaustion. As illustrated in **Scheme** **1**, the “nanoenabled energy interrupter” exhibits multifunctional characteristics, including i) active targeting ability to tumor cells, leading to the accurate reorganization and efficient endocytosis of NPs; ii) the disaggregation of zinc cores was collectively controlled by a hyaluronidase (HAase)‐responsive gate and pH‐sensitive gate, achieving a specifical “Zn^2+^ interference” effect of tumor, leading to the decrease of NAD^+^ and inactivation of GAPDH, obtaining strong glycolysis inhibiting; iii) “Zn^2+^ interference” can specifically activate the catalytic shearing effect of GD for decreasing GLUT1 expression, cutting off glucose supply, further realizing systematic energy exhaustion in tumor. By combination of triple‐characteristics, the dual gate‐controlled “nanoenabled energy interrupter” achieved potent starving efficacies against melanoma in a mutually reinforcing way, while showed a relatively low impact on normal melanocytes.

## 2 Results

### 2.1 Synthesis and Characterization of “Nano‐enabled Energy Interrupter”

**Figure 1:**
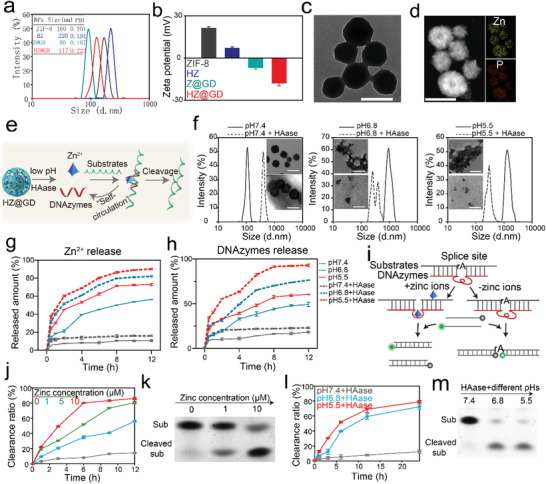
a,b) Size distribution a) and *ζ* potential b) of ZIF‐8, HZ (HA‐modified ZIF‐8), Z@GD (ZIF‐8 encapsulating GLUT1 DNAzyme) and HZ@GD (HA‐modified ZIF‐8 encapsulating GLUT1 DNAzyme) (*n* = 3). c) Representative TEM image of HZ@GD showing that HZ@GD nanoparticles are synthesized with a homogeneous size distribution about 76 nm. Scale bar, 100 nm. d, Elemental mapping of Zn, P in HZ@GD reflecting that Zn^2+^ and DNAzymes are successfully presented in HZ@GD, Scale bar, 100 nm. e) The schematic diagram of pH and HAase‐controlled release of DNAzyme and the corresponding Zn^2+^ from HZ@GD for efficient mRNA substrate cleavage. f) Representative size distribution and TEM images of HZ@GD in PBS at pH 7.4, 6.8, and 5.5 with or without HAase (0.2 mg mL^−1^), describing the degradation degree of HZ@GD under different conditions. Scar bar, 100 nm. The embedded figures are TEM images. Among these, the upper is HZ@GD under different pH, while the below is HZ@GD under different pH with HAase (0.2 mg mL^−1^). g,h) In vitro Zn^2+^ g) and DNAzymes h) release curves of HZ@GD in PBS at pH 7.4, 6.8, and 5.5 with or without HAase (0.2 mg mL^−1^), (*n* = 3). i) The principle of fluorescencenano assay for detecting DNAzymes shear efficacy. The fluorescence intensity is the evaluation criteria of DNAzymes shear efficacy. The higher the fluorescence intensity, the higher the shear efficiency. j,k) Cleavage kinetics analysis j) and PAGE analysis k) of DNAzymes‐mediated cleavage efficacy under different concentrations of Zn^2+^ (*n* = 3). The clearance ration is calculated based on the following formula: The clearance ration = RLU_(reaction time points)_/RLU_(0 h)_ X100% l, m, Cleavage kinetics analysis l) and PAGE analysis m) of HZ@GD‐mediated cleavage efficiency under different pHs with 0.2 mg mL^−1^ HAase (*n* = 3). All data are shown as the Mean ± SD from three independent experiments.

In this work, a highly efficient Zn^2+^‐activated mRNA‐cleaving DNAzyme sharing a common motif with the “8‐17” deoxyribozyme was selected and transformed into a therapeutic DNAzyme that can specifically recognize and digest intracellular GLUT1 mRNA for down‐regulate GLUT1 expression (Figure S1, Supporting Information). By embedding GLUT1 mRNA‐cleaving DNAzyme (GD) into nanosized ZIF‐8 and then tethered by HA, the final nanostructures (HZ@GD) were successfully synthesized and the detailed experimental steps were provided in the Experimental Section of the Supporting information. ZIF‐8, HA‐modified ZIF‐8 (HZ), and ZIF‐8 embedded with GLUT1 mRNA‐cleaving DNAzyme (Z@GD) were also synthesized based on the similar procedure method to HZ@GD. First, physicochemical characterizations reflected that the HZ@GD possessed a lower hydrodynamic diameter of 117 nm than that of ZIF‐8 (160 nm) and HZ (220 nm) nanoparticles (**Figure** **1**a), which attributed to the faster nucleation originating from the intense coordination interaction between Zn^2+^ and DNAzymes.^[^ ^17^ ^]^ Compared with the zeta potential of ZIF‐8 (28.5±1.5 mV), HZ (8.9±1.4 mV), and Z@GD (−6.8±1.2 mV), the zeta potential of HZ@GD (−17.9±1.9 mV) was obviously decreased after DNAzyme encapsulation and HA coating (Figure 1b). Transmission electron microscopy (TEM) visualization displayed uniform spherical morphology of HZ@GD, ZIF‐8, HZ, and Z@GD (Figure 1c; and Figure S2, Supporting Information). Element mapping showed that Zn elements (from ZIF‐8) and phosphorus (from DNAzyme) distributed homogeneously in HZ@GD (Figure 1d). Meanwhile, the loading efficiency of DNAzymes was determined to be ≈81.5% (Figure S3, Supporting Information). Owing to the coordination between the carboxyl group of HA and Zn^2+^, HA was successfully coated on the surface of ZIF‐8 and the modification rate of HA was calculated to be 3.27% by TGA analysis (Figure S4, Supporting Information). The surface coating of HA ensured the stability of HZ@GD in physiological fluids (PBS, saline and cell medium) as evidenced by the stable hydrodynamic sizes within one week (Figure S5, Supporting Information). Afterward, powder X‐ray diffraction (PXRD) pattern verified the as‐prepared HZ@GD was typical ZIF‐8 phases (Figure S6, Supporting Information). The above results demonstrated that HZ@GD nanoparticles were successfully prepared.

It has been documented that Zn‐N bonds are sensitive to a weakly acidic environment,^[^ ^18^ ^]^ which can dissociate and enable “Zn^2+^ extraction” from ZIF‐8. Thus it is expected that the “Zn^2+^ extraction” and DNAzymes release from the “nanoenabled interrupter” can be initiated by a pH‐sensitive gate (Figure 1e). Additionally, HA, as a coating of the “nanoenabled interrupter,” is also a hyaluronidase (HAase)‐sensitive gatekeeper for controlled drug release. Therefore, the Zn^2+^and DNAzymes (labeled with AF488) release characteristics of HZ@GD jointly driven by low pH and HAase were characterized by ICP‐MS assay and fluorescence analysis, respectively. First, we verified the pH‐mediated skeleton collapse of ZIF‐8 by UV–vis spectrum (Figure S7, Supporting Information), reflecting the acidic sensitivity of ZIF‐8. Owing to the pH‐responsive capability, both Zn^2+^ and DNAzymes were efficiently released from Z@GD under pH 6.8 or 5.5 and the release rates were faster than that under a physiological environment (pH = 7.4) (Figure S8, Supporting Information). In contrast, the addition of HAase in these solutions had a negligible effect on GD release from Z@GD. For HZ@GD. TEM images allowed us to observe a more complete structural collapse of HZ@GD in acidic pH solution containing HAase, while, owing to the absence of HAase, the structural dissociation of HZ@GD was incomplete with pH descends (Figure 1f). The results of dynamic light scattering (DLS) also confirmed the dual stimulation‐responsive structure disassemble of HZ@GD (Figure 1f). Meanwhile, the release curves further showed that HA coating could significantly decrease the release rates of both Zn^2+^ and DNAzymes in a neutral environment (Figure 1g,h). Benefiting from the HA degradability induced by HAase and pH sensitivity of ZIF‐8, higher release rates of Zn^2+^ and DNAzymes from HZ@GD were found in an acidic environment containing HAases than that in the single acidic environment or HAase‐containing environment (Figure 1g,h). Differing from normal cells, tumor cells feature endogenous weak acidification and high HAase levels.^[^ ^19^ ^]^ Therefore, such a dual gate (pH and HAase)‐controlled drugs release peculiarity is expected to guarantee that the HZ@GD‐mediated operation of Zn^2+^ overloading and DNAzymes release occurs specifically and efficiently within tumor cells. Importantly, it is worth further analysis that whether the concomitantly released Zn^2+^ can supply an adequate amount of DNAzyme cofactor to perform the biocatalytic operation. Therefore, we first detected the mRNA digestion efficiency of DNAzymes in the presence of Zn^2+^ by fluorescence analysis and polyacrylamide gel electrophoresis, respectively. To facilitate the operation in vitro, we used a DNA strand (Figure S1, Supporting Information) containing an rA base (where rA is ribonucleotide base and is cleavage site), and sharing the same GLUT1 mRNA sequence, as a simulative substrate to verify the cleavage efficiency. For real‐time monitoring of the shearing ability, the assay of fluorescence‐resonance energy transfer (FRET) was conducted, and the experimental principle was depicted in Figure 1i. As expected, the substrate‐cleaving effect of DNAzymes showed strong Zn^2+^ concentration‐dependent and incubation time‐dependent properties (Figure 1j). The result of polyacrylamide gel electrophoresis further reflected that 1 × 10^−6^ m Zn^2+^ is fully sufficient to initiate the operation of DNAzymes (Figure 1k). Notably, the amount of released Zn^2+^ from HZ@GD under pH 6.8 containing 0.2 mg mL^−1^ of HAase was calculated about 19.5 × 10^−6^ m after 4 h treatment (Figure 1b), which is fully capable of turning on the cleavage activity of DNAzymes. Next, we further assessed the catalytic ability of HZ@GD to cleave GLUT1 mRNA by fluorescence analysis and polyacrylamide gel electrophoresis, respectively. As shown in Figure 1l,m, ≈63% and 72% of substrate were cleaved by HZ@GD in the 0.2 mg mL^−1^ HAase‐containing PBS (pH 6.8 and 5.5) after 20 h of treatment, while negligible scavenging activity of DNAzymes from HZ@GD was found in HAase‐containing physiological environment.

### 2.2 Identification of Tumor‐Targeting and Lysosomal Escape Ability of HZ@GD

**Figure 2:**
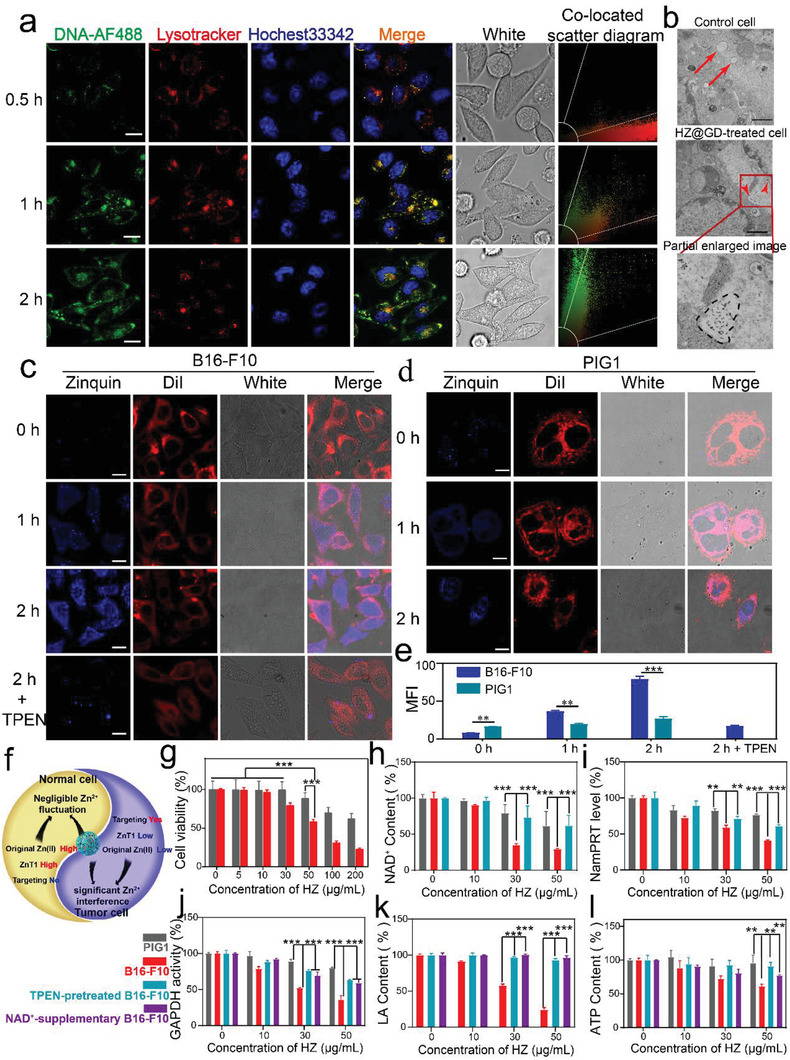
The selective disrupting intracellular Zn^2+^ homeostasis for inhibiting energy metabolism on melanoma. a) Representative CLSM images of biodistribution of AF488‐labeled HZ@GD in endo/lysosomes for different time treatment to B16‐F10 cells. The abscissa of the colocated scatter diagram indicated red fluorescence (lysosome), and the ordinate of the colocated scatter diagram indicated green fluorescence (DNA‐AF488). Hochest33342 was used to stain cell nucleus. Scale bar: 25 µm. b) Bio‐TEM images of B16‐F10 cells or HZ@GD‐treated B16‐F10 cells. Untreated B16‐F10 cells were used as control. Scale bar: 1 µm; Red arrows pointed to lysosomes. The black dotted frame pointed to the broken lysosome. c,d) CLSM images of intracellular free Zn^2+^ production after treatment with HZ on B16‐F10 c) pretreated without or with TPEN and PIG1 cells d) for 0, 1, and 2 h, respectively. Blue (zinquin) indicated intracellular free Zn^2+^. Red (DiI) indicated cell membrane. Scale bar: 25 µm. e) MFI (Mean Fluorescence Intensity) of free Zn^2+^ (blue fluorescence) in the fluorescence images, *n* = 3. f) Illustration of selective Zn^2+^ overloading for energy metabolism inhibition of tumor, showing that the potential reasons of preferential Zn^2+^ overloading in B16‐F10 cells than PIG1 cells. g) Cell viability of B16‐F10 cells and PIG1 cells subjected to HZ (*n* = 6). h–l) NAD^+^ h), NamPRT i), GAPDH activity j), secretory LA k), and ATP levels l) in B16‐F10 (without or with pre‐treated TPEN or NAD^+^) or PIG1 cells treated with different concentrations of HZ (*n* = 3). Results are presented as means ± s.d. *P*‐values were determined by a two‐tailed unpaired *t*‐test. *P < 0.05, **P < 0.01, ***P < 0.001, ****P < 0.0001.

Next, to investigate whether HZ@GD owned specific tumor‐targeting ability, we compared the uptake differences of nanoparticles between B16‐F10 cells (malignant melanoma cells) and PIG1 cells (melanocytes) by tracking the fluorescence of DNAzyme via confocal laser scanning microscopy (CLSM) and flow cytometer, respectively. As shown in Figure S9a (Supporting Information), compared with PIG1 cells, more green fluorescence could be seen in B16‐F10 cells, indicating a more efficient nanoparticles internalization in tumor cells. Meanwhile, an obvious decrease in the uptake of HZ@GD was found in B16‐F10 cells pretreated with anti‐CD44 antibody than that in PIG1 cells, suggesting that CD44‐mediated actively targeting mechanism facilitates the uptake of HZ@GD for tumor cells. The result of flow cytometer also presented a similar uptake trend (Figure S9b,c, Supporting Information). After successful tumor internalization, the lysosomal escape associated with HZ@GD was further assessed by transfecting with nanoparticles labeled with carboxyfluorescein (AF488) at different time points (0.5, 1, and 2 h). Previous studies have demonstrated that the lysosomal escape of HZ@GD is initiated by the protonation of the imidazole ring,^[^ ^18^ ^]^ which is followed by the release of Zn^2+^ and DNAzymes to the cytoplasm. CLSM images reflected that HZ@GD localized in lysosomes at 0.5 h of incubation, validating by the overlap fluorescence between green fluorescence (DNAzyme) and red fluorescence (lysosome). In contrast, 1 and 2 h post‐transfection presented effective separation between DNAzymes and lysosomes, indicating the subsequent release of Zn^2+^ and DNAzymes into the cytoplasm (**Figure** **2**a). More importantly, Bio‐TEM images intuitionistically reflected the ruptured lysosome of HZ@GD‐treated B16‐F10 cells and the nanoparticles leaked from lysosomal lumen (Figure 2b), which verified the successful lysosomal escape of nanoparticles.

### 2.3 HZ Triggers Preferential Intracellular Zn^2+^ Interference on Malignant Melanoma Cells

Subsequently, whether HZ could result in intracellular Zn^2+^ overloading was tested. First, ICP‐MS was used to detect Zn^2+^ concentrations in B16‐F10 cells. As shown in Figure S10 (Supporting Information), B16‐F10 cells treated with HZ nanoparticles exhibited a noticeable increase in Zn^2+^ concentration with up to the micromole level. While, in normal cells (PIG1), HZ triggered inconspicuous fluctuations in Zn^2+^ concentrations, hinting that HZ resulted in a specifical intracellular Zn^2+^ accumulation in tumor cells. It is worth mentioning that the intracellular accessible Zn^2+^ are only in the picomolar range,^[^ ^13a^ ^]^ such a prominent intracellular Zn^2+^ accumulation in tumor triggered by HZ NPs is sufficient for activating DNAzymes. Moreover, taking advantage of a free Zn^2+^ indicator (FluoZin‐3 probe), intracellular free Zn^2+^ levels were further visualized in B16‐F10 cells and PIG1 cells via CLSM and flow cytometer, respectively (Figure 2c–e; and Figure S11, Supporting Information). As expected, the B16‐F10 cells coincubated with HZ showed a higher fluorescence intensity in comparison with the PIG1 cells, indicating a higher intracellular accumulation of free Zn^2+^ in tumor cells, which was consistent with the ICP‐MS results. In addition, TPEN, a specific Zn^2+^ chelator, attenuated the blue fluorescence induced by HZ, which confirmed that the increased fluorescence intensity is caused by the accumulation of free Zn^2+^. Meanwhile, time‐lapse imaging in the single B16‐F10 cell also further provided a powerful tool to visually observe intracellular Zn^2+^ accumulation triggered by HZ in real‐time (Figure S12, Supporting Information).

Above all, we found that HZ could induce a specific Zn^2+^ accumulation in tumor cells, the underlying mechanisms deserved further exploration. Of course, HA‐mediated CD44 targeting mechanisms are mainly responsible for selective intracellular Zn^2+^ overloading. As expected, A higher expression level of CD44 protein was found on B16‐F10 cells than that on PIG1 and HL‐7702 cells (Figure S13a,b, Supporting Information), which guarantees the excellent HA‐mediated tumor targeting capability of HZ. Notably, there is a significant difference in the basic Zn^2+^ levels between B16‐F10 cells and PIG1 cells (Figure 2c–e; and Figure S10–S11, Supporting Information), the original Zn^2+^ content of B16‐F10 cells only had one‐fifth of the content of PIG1 cells. To determine whether such differences of original Zn^2+^ level also contribute to selective Zn^2+^ overloading, we compared the difference of accumulated Zn^2+^ level between B16‐F10 cells and PIG1 cells with the same uptake amount of HZ. Interestingly, although there is the same degree of HZ uptake (B16‐F10 cells incubation with 50 µg mL^−1^ HZ‐FITC for 1 h, PIG1 cells incubation with 80 µg mL^−1^ HZ‐FITC for 1 h, see Figure S14, Supporting Information), the fluctuation degree of intracellular Zn^2+^ was still higher in B16‐F10 cells than that in PIG1 cells (Figure S15, Supporting Information).

To prove the generality of the result, another normal cell, HL‐7702 cells (hepatocytes) was selected as control cells because the liver is the largest metabolic organ in vivo for metabolizing most nanoparticles. As expected, in the case of the same intake of nanoparticles (Figures S14 and S16a, Supporting Information), HZ still triggered a more obvious Zn^2+^ accumulation in B16‐F10 cells compared with HL‐7702 cells, although the original Zn^2+^ amounts of HL‐7702 cells are higher than B16‐F10 cells (Figure S16b,c, Supporting Information). By comparison, Zn^2+^ homeostasis of tumor cells could be more easily disturbed than normal cells (PIG1 and HL‐7702 cells), one of the potential reasons is the inherently low Zn^2+^ levels in B16‐F10 cells. The inference is also supported by the previous research of Costello et al.^[^ ^12^ ^]^ In addition, previous studies reported that ZnT1, a Zn^2+^ transfer protein that functions to reduce cytoplasmic Zn^2+^ concentrations, is found to be downregulated in tumor cells, leading to weak Zn^2+^ efflux capacity of tumor cells,^[^ ^10^, ^20^ ^]^ which may be another potential mechanism of selective Zn^2+^ overloading. In summary, “nanoenabled energy interrupter” could selectively induce Zn^2+^ overloading in tumor cells, the underlying mechanism is the difference of the original Zn^2+^ levels and expression of Zn^2+^ efflux protein ZnT1 between tumor cells and normal cells, as well as tumor cell targeting effect of HA (Figure 2f).

### 2.4 Zn^2+^ Interference Significantly Inhibits Glycolytic Pathway In Malignant Melanoma Cells

Any abnormal changes of intracellular Zn^2+^ concentration would influence cellular processes and signaling pathways, eventually, impair cell activity. First, the hypothesis was evidenced by a simple incubation of free Zn^2+^ with B16‐F10 cells, which presented a sharp decrease in cell viability, and an decrease of cytotoxicity on TPEN‐pretreated B16‐F10 cells after treatment with free Zn^2+^ (Figure S17a, Supporting Information). Simultaneously, HZ showed markedly obvious inhibition of the viability of B16‐F10 cells in a dose‐dependent manner (half‐maximal inhibitory concentration (IC_50_): 53.2 µg mL^−1^) (as depicted in Figure 2g). While incubation with dimethylimidazole (another degradation product of ZIF‐8) alone was harmless to B16‐F10 cells (Figure S17a, Supporting Information), indirectly demonstrating HZ‐mediated Zn^2+^ overloading‐induced cell death. Satisfactorily, normal cells (PIG1 cells) managed to tolerate the adverse influence of HZ with much higher cell viabilities (IC_50_: 461.8 µg mL^−1^) than tumor cells (as depicted in Figure 2g). It is worth mentioning that this higher tolerance of normal cells was attributed to the inefficient Zn^2+^ overloading. Studies suggested that loss of cellular energy may be the main “culprits” for Zn^2+^ overloading toxicity.^[^ ^21^ ^]^ For energy metabolism, nicotinamide adenine dinucleotide (NAD^+^) is a key redox factor and substrate in ATP production pathway, and has been identified as a promising target of energy deprivation for tumor therapy, we therefore tested that whether Zn^2+^ overloading could specifically block the production of NAD^+^ in tumor cells, resulting in a loss of cellular energy. First, we checked the level of NAD^+^ in B16‐F10 cells and PIG1 cells with HZ treatments, respectively. As shown in Figure 2h, compared with the control group, there was a 65.1% and 71.0% reduction of NAD^+^ in B16‐F10 cells subjected to HZ with different concentrations of HZ treatment (30 and 50 µg mL^−1^), respectively. In sharp contrast, the intracellular NAD^+^ levels showed no significant change in PIG1 cells. In addition to the decrease of NAD^+^, we also found that nicotinamide phosphoribosyltransferase (NamPRT), the rate‐limiting and most crucial NAD^+^ biosynthetic enzyme,^[^ ^22^ ^]^ was decreased in HZ treated B16‐F10 cells. On the contrary, HZ treatment resulted in a negligible impact on NamPRT level of PIG1 cells (Figure 2i). Meanwhile, TPEN effectively reversed the effects of HZ on concentrations reduction of both NAD^+^ and NamPRT of B16‐F10 cells (Figure 2h,i). These results suggested that intracellular Zn^2+^ overloading harmed the pathways of NAD^+^ generation. Indeed, reduced NAD^+^ could restrict the activity of the NAD^+^‐dependent enzymes, such as reduced glyceraldehyde‐phosphate dehydrogenase (GAPDH), an enzyme that is required for glycolysis pathway.^[^ ^23^ ^]^ Therefore, the activity of GAPDH in B16‐F10 cells and PIG1 cells subjected to HZ was tested by a GAPDH activity assay kit, respectively. Similar to the decreasing trend of NAD^+^, the activity of GAPDH was obviously inhibited in HZ concentration‐dependent manner in B16‐F10 cells compared with PIG1 cells (Figure 2j).

Moreover, abnormal proliferation and microenvironment of tumor result in the differences of metabolic mechanisms between tumor cells (Aerobic glycolytic pathway) and normal cells (TCA, Tricarboxylic acid pathway),^[^ ^24^ ^]^ which made tumor cells are more dependent on the energy produced by the glycolysis pathway. Next, two typical energy products of aerobic glycolysis, lactic acid (LA) and ATP, were monitored in B16‐F10 cells. As expected, after HZ incubation, a distinct decrease of LA levels was detected in B16‐F10 cells (Figure 2k). Subsequently, as shown in Figure 2l, B16‐F10 cells cultured with HZ displayed a significant reduction of the cellular ATP levels. Furthermore, the pretreatment of TPEN and NAD^+^ in B16‐F10 cells also effectively reversed the effects of HZ on energy blockade (Figure 2j–l). To further validate the pivotal role of Zn^2+^ overloading induced by HZ in energy loss, we also monitored the change of ATP level on B16‐F10 cells after incubated HZ, free Zn^2+^ and dimethylimidazole, respectively. As expected, free Zn^2+^ and HZ treatment showed a similar inhibition trend of ATP levels on B16‐F10 cells, while dimethylimidazole did not contribute to the loss of ATP on B16‐F10 cells (Figure S17b, Supporting Information). Moreover, smililar with HZ treatment (Figure 2l), when the excessive free Zn^2+^ were inhibited by the zinc chelator TPEN, the Zn^2+^ overloading‐induced ATP loss was reduced by more than one‐third (Figure S17b, Supporting Information). All these results demonstrated that the role of Zn^2+^ was important to the HZ‐induced energy loss procedure. Of note, owing to ineffective Zn^2+^ overloading and differences in energy metabolic pathways, the remarkable ATP decrease effect of HZ was not shown on PIG1 cells (Figure 2l). Meanwhile, normal hepatocytes (HL‐7702 cells) are also used as a control to further evaluate the ability of energy inhibition mediated by HZ. As exhibited in Figure S16d–f (Supporting Information), HZ resulted in the preferential B16‐F10 cells energy exhaustion compared with HL‐7702 cells, thus verifying the effectiveness of preferential Zn^2+^ overloading strategy.

### 2.5 Influence of Zn^2+^ Interference on Intracellular mRNA

**Figure 3:**
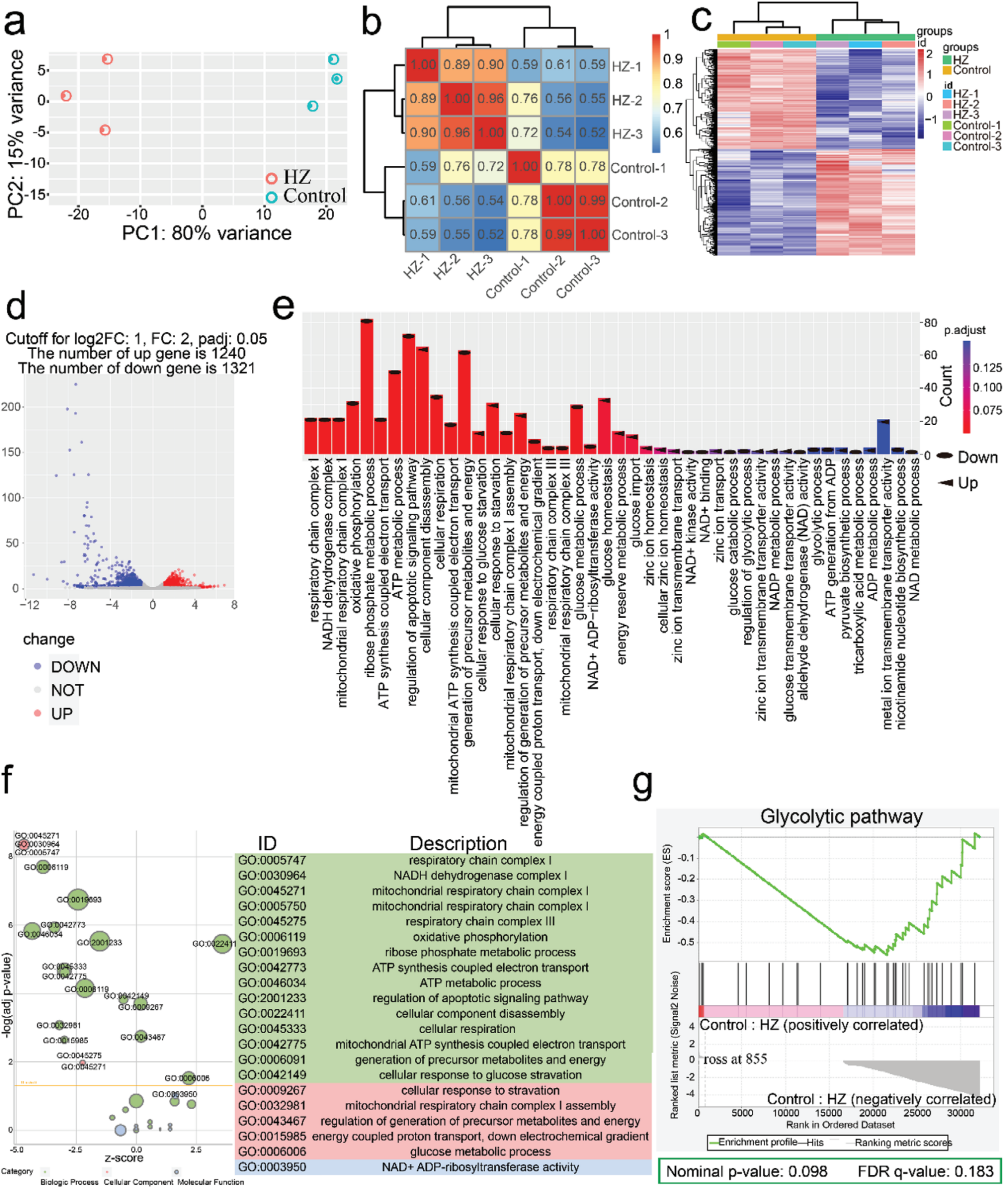
RNA sequencing analysis on B16‐F10 cells subjected to HZ treatment. a) the diagram of principal component analysis (PCA), the ordinate is the first principal component and the abscissa is the second principal component. b) Heat map of intersample correlation. The horizontal and vertical coordinates in the figure are the square of the correlation coefficients of each sample. c) Clustering heat map of differentially expressed genes (DEGs). The abscissa is the sample name, and the ordinate is the normalized value of the DEGs. The redder the color, the higher the expression level, and the bluer the expression level, the lower the expression level. d) Volcano map of DEGs. The up‐regulated genes are represented by red dots and down‐regulated genes by blue dots. e,f) GO enrichment analysis e) and Go plot analysis f) of DEGs between HZ and control treatment group. g) gene set enrichment analysis (GSEA) for determining the up‐regulation or down‐regulation of the glycolytic pathway in HZ‐treated B16‐F10 cells.

To further explore the multilevel energy deprivation mechanisms caused by nano‐enabled “Zn^2+^ interference”, RNA sequencing of B16‐F10 cells after treatment with HZ NPs was implemented, and untreated B16‐F10 cells were used as control. Principal component analysis (PCA) indicated that sample data within the same treatment group were clustered together and the sample data between two treatment groups were scattered, reflecting the high quality of the RNA sequencing data (**Figure** **3**a). The result of the intersample correlation heat map visually showed the sample differences between groups and the sample duplication within groups. As expected, a high correlation (*r* ^2^>0.8) within the same treatment group was found, but no significant correlations between control treatment and HZ treatment (Figure 3b), further confirming that the gene differences were specifically caused by the treatment with HZ. The transcriptomic profile of sorted B16‐F10 cells was assessed by RNA‐sequencing and reads of each sample were mapped on 21 849 protein‐coding genes from the reference genome. Differential expression analysis verified 2561 differentially expressed genes (DEG) between the control sample and HZ sample (Figure 3c). The result of volcano plots further revealed that, of the 2561 DEGs, 1240 were upregulated and 1321 were downregulated in HZ‐treated B16‐F10 cells (Figure 3d). Gene Ontology (GO) analysis of DEGs showed that the vast majority of significantly enriched functions correspond to enrichments in downregulated genes (Figure 3e). A large group of those belongs to metabolism pathway, implying an overall decrease in energy metabolic activities, some of them being typically associated with NAD^+^ biosynthesis, ATP production (e.g., aldehyde dehydrogenase (NAD) activity, oxidative phosphorylation and ATP synthesis coupled electron transport). Of interest, up‐regulated gene‐sets also unveil an important enrichment in intracellular Zn^2+^ regulation functions (e.g., zinc ion homeostasis and zinc ion transmembrane transport), which may point to intracellular Zn^2+^ fluctuation triggered by HZ NPs. Meanwhile, Kyoto Encyclopedia of Genes and Genomes (KEGG) pathway analysis (Figure S18, Supporting Information) further revealed that HZ treatment significantly disrupted the metabolic process of B16‐F10 cells, including oxidative phosphorylation, purine metabolism, pyrimidine metabolism, glutathione metabolism, fatty acid metabolism, and nicotinate and nicotinamide metabolism, et. al. Next, go plot analysis also demonstrated that the activities of most energy metabolism pathways were hindered in HZ‐treated B16‐F10 cells (Figure 3f). Importantly, gene set enrichment analysis (GSEA) showed that there is a negative correlation between GEs and glycolysis pathway, hinting at an overall downward trend in glycolysis pathway in B16‐F10 cells after treated with HZ NPs (Figure 3g). Above all, all results demonstrated that HZ blocked the proliferation of B16‐F10 cells, which could be attributed to multilevel energy loss caused by nanoenabled “Zn^2+^ interference.”

### 2.6 HZ@GD Induces Systematic Energy Exhaustion on Malignant Melanoma Cells

**Figure 4:**
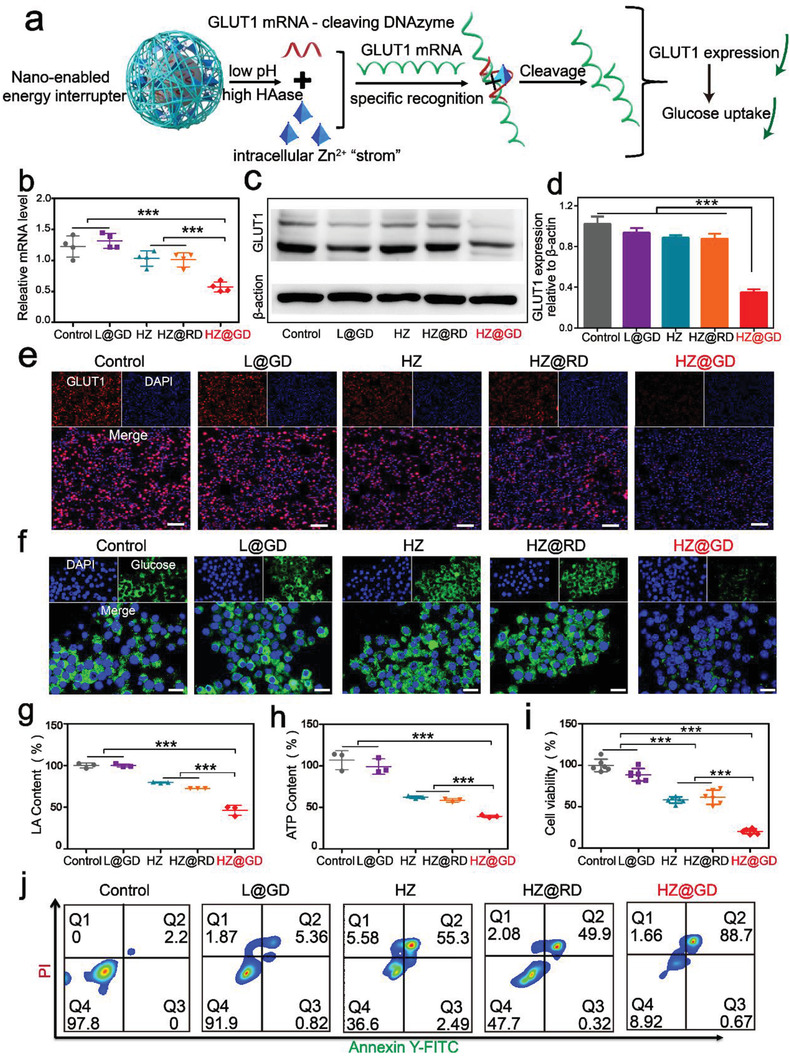
HZ@GD induces systematic energy exhaustion on malignant melanoma cells. a) Scheme illustration of “nanoenabled energy interrupter” for self‐activated gene silencing therapy. b) qRT‐PCR assay for monitoring GLUT1 mRNA expression in B16‐F10 cells with various treatments for 12 h, including untreated, L@GD, HZ, HZ@RD, and HZ@GD treatments (*n* = 4). c–e) Western blot assay c), semiquantitative analysis d) (*n* = 3) and Immunofluorescence analysis e) of GLUT1 protein in B16‐F10 cells with different treatments for 12 h. Scale bar: 100 µm. In immunofluorescence assay, GLUT1 was pseudocolored as red and cell nucleus was pseudocolored as blue. f) Immunofluorescence analysis of intracellular glucose levels in B16‐F10 cells subjected to different formulations for 12 h, Scale bar: 100 µm. Glucose was pseudocolored as green and cell nucleus was pseudocolored as blue. g,h) LA g) and ATP h) levels in B16‐F10 cells treated with different formulations for 12 h (*n* = 3). i,j) Cell viability i) (*n* = 6) and apoptosis and necrosis analysis j) (*n* = 3) after treatment with various formulations on B16‐F10 cells for 24 h. Results are presented as means ± s.d. *P*‐values were determined by a two‐tailed unpaired *t*‐test. *P < 0.05, **P < 0.01, ***P < 0.001, ****P < 0.0001.

Blockade of intracellular glycolytic pathway often leads to the adaptive upregulation of the glucose demand, promoting the upregulation of glucose transporters 1 (GLUT1) expression in tumors.^[^ ^14^ ^]^ Therefore, to achieve exhaustive energy deprivation, an imperious demand has been raised for further GLUT1 blockade. Interestingly, tumor‐targeted nanoenabled “Zn^2+^ interference” provided an ingenious means for specifical cutting off energy sources via Zn^2+^‐activating GLUT1 mRNA down‐regulation. As illustrated in **Figure** **4**a, HZ@GD could spontaneously release GLUT1 DNAzyme and adequate Zn^2+^ under the lysosomal environment. Besides using as an NAD^+^ inhibitor, Zn^2+^ also served as the cofactor to activate GLUT1 DNAzyme for GLUT1 gene silencing, ultimately cutting off glucose supply. To verify this, the cleavage of GLUT1 mRNA in B16‐F10 cells cultured with HZ@GD was first evaluated via qRT‐PCR (quantificational real‐time polymerase chain reaction). Simultaneously, different formulations including L@GD (Liposome@GLUT1 DNAzyme), HZ@RD (HA/ZIF‐8@Random sequence DNAzyme, which lack of specific shear sequence of GLUT1 mRNA), and HZ were used as the control groups. Figure 4b displayed that the expression of GLUT1 mRNA was significantly down‐regulated in B16‐F10 cells incubated with HZ@GD, while it barely changed in other groups. Although there was little difference between HZ@GD and L@GD in the cellular uptake (Figure S19, Supporting Information), a lower cleavage efficiency was found in L@GD‐treated cells compared to HZ@GD group, since liposome could not activate DNAzyme due to the lack of cofactors supplement. The dramatically improved GLUT1 mRNA‐cleavage ability of “nanoenabled energy interrupter” may be attributed to specific Zn^2+^ overloading in tumor cells. The results of western blotting showed that the levels of GLUT1 were significantly down‐regulated in B16‐F10 cells with HZ@GD treatment (Figure 4c,d). Additionally, an immunofluorescence assay was also carried out to further verify the ability of HZ@GD‐mediated GLUT1 downregulation. B16‐F10 cells subjected to HZ@GD exhibited weaker red fluorescence signal than those control groups (Figure 4e), implying that the expression of GLUT1 protein was effectively inhibited. Furthermore, a quantitative glucose uptake experiment was carried out for estimating the exact inhibition efficacy of HZ@GD. It could be clearly seen in Figure S20 (Supporting Information), the level of intracellular glucose after treatment of HZ@GD was decreased by 63% compared with the untreated group, while no obvious change in other groups. CLSM images also revealed that HZ@GD could efficiently restrict glucose into the cells (Figure 4f). Overall, these results confirmed that “nanoenabled energy interrupter” successfully blocked intracellular energy sources via Zn^2+^‐mediated self‐activated gene silencing. As a “nanoenabled energy interrupter,” HZ@GD was used to thoroughly inhibit the energy metabolism pathway of malignant melanoma by combining inhibition of downstream link in glycolysis with blockade of upstream energy supply. The suppressive ability of “nanoenabled energy interrupter” in metabolic pathway was determined with the secretory LA and ATP levels in B16‐F10 cells. As presented in Figure 4g, L@GD group showed a negligible impact on secretory LA levels in B16‐F10 cells, while decreasing by 55.0%, 20.0%, and 19.9% in HZ@GD, HZ@RD, and HZ groups, respectively. Similarly, ATP levels in HZ@GD group (39.3%) displayed a significant decrease than that of HZ@RD and HZ groups (58.7% and 67.2%), respectively (Figure 4h). After confirming the superior ability of energy inhibition, we further examined the antitumor potential of HZ@GD. Owing to the Zn^2+^‐mediated dual function, HZ@GD exhibited the strongest inhibition on B16‐F10 cells than other groups (Figure 4i). Moreover, the apoptotic ratio of HZ@GD increased to 91.53%, which was much higher than HZ@RD (49.87%), HZ (51.73%), and L@GD (4.54%) (Figure 4j; and Figure S21, Supporting Information). Notably, although the DNAzyme targeting GLUT1 mRNA of HL‐7702 cells was obtained (Figure S22, Supporting Information), the cytotoxicity of HZ@GD on HL‐7702 cells was far less than B16‐F10 cells (Figure S23, Supporting Information), suggesting the preferential inhibition of “nanoenabled energy interrupter” on melanoma cells. Meaningfully, systemic energy blockade not only inhibits the tumor growth, but also is expected to facilitate the obstruction of tumor metastasis.^[^ ^3^ ^]^ As for the tumor metastasis, “Zn^2+^ interference”‐mediated starvation strategy can serve as an alternative method to address the tricky dilemma via the “top‐down” energy blockade. Thus, future studies will further focus on designing a more optimized nanosystem with robust Zn^2+^ interference ability for tackling the existing conundrums of tumor metastasis and exploring ion‐induced novel treatment models.

### 2.7 HZ@GD Inhibits Tumor Growth via Systematic Energy Exhaustion

**Figure 5:**
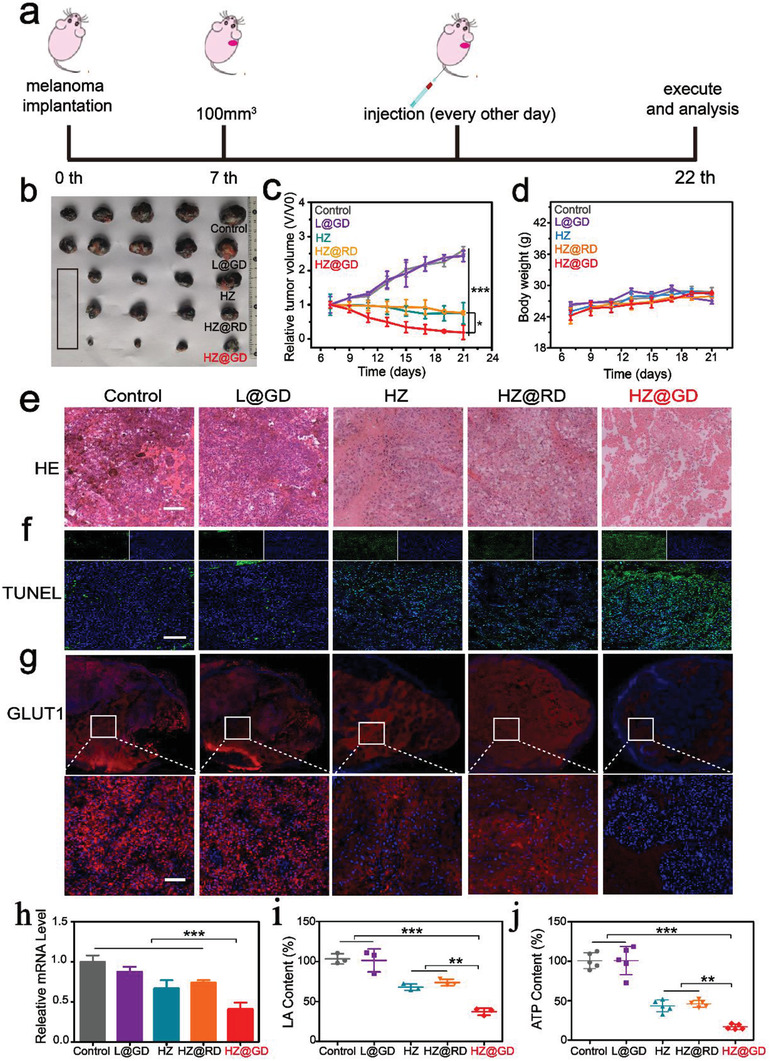
HZ@GD inhibits tumor growth via systematic energy exhaustation. a) Schedule of therapeutic process of “nanoenabled energy interrupter.” Injection of nanoformulations was conducted every other day for a total of 14 days. b) Photos of tumor tissues collected from different groups on the 22nd day. c,d) Tumor growth profiles c) and Bodyweight change profiles d) recorded during 22 days (*n* = 5). e,f) Representative images of HE staining e) and TUNEL staining f) in tumor tissues after treatment of various formulations (scale bar: 100 µm). g,h) Immunostaining g) of GLUT1 protein (red: GLUT1; blue: DAPI; scale bar: 100 µm) and qRT‐PCR analysis h) of GLUT1 mRNA expression in tumor tissues after treated with different formulations (*n* = 4). i,j) Intratumoral LA i) and ATP levels j) after treated with different formulations (*n* = 3). Results are presented as means ± s.d. *P*‐values were determined by a two‐tailed unpaired *t*‐test. *P < 0.05, **P < 0.01, ***P < 0.001, ****P < 0.0001.

Inspired by the prominent performance in vitro, the therapeutic efficiency of “nanoenabled energy interrupter” in vivo was further explored. After ensuring the excellent hemocompatibility (Figures S24 and S25, Supporting Information), the in vivo biodistribution of HZ@GD was supervised by the fluorescence of HA/ZIF‐8@IR783, which IR783 was encapsulated into HZ. Profiting from the HA‐mediated tumor targeting ability, the retention ability of HA/ZIF‐8@IR783 in tumor site was stronger than that of free IR783 and ZIF‐8@IR783 (Figure S26a–c, Supporting Information), which provided requisite precondition for “nano‐enabled energy interrupter”‐induced antitumor therapy. For further exploration, ICP‐MS was carried out to quantitatively analyze the biodistribution of Zn^2+^. As expected, HZ@GD could be also effectively enriched in tumor sites (Figure S27, Supporting Information). Subsequently, the released Zn^2+^ in the tumor site were visualized by zinquin staining (Figure S28, Supporting Information), indicating the superior ability of “nanoenabled energy interrupter”‐induced intratumoral Zn^2+^ accumulation. And then the antitumor effect of “nanoenabled energy interrupter” in vivo was evaluated via B16‐F10 tumor‐bearing C57BL/6 mice and the therapeutic schedule was shown in **Figure** **5**a. HZ@GD significantly decreased the volume of tumor, and the inhibition rate of tumor growth was up to 80.8%, while tumor inhibition rate of HZ and HZ@RD groups was 25.2% and 23.6%, respectively (Figure 5b,c). In addition, the safety of all these administrated treatments was assessed by the increasing fatness (Figure 5d). After treatment, the mice were executed and the major organs were harvested to carry out a series of experiments for further assessing therapeutic effect. HE images showed large amounts of karyopyknosis and the severer structural deformation in HZ@GD treated group (Figure 5e), while there is negligible damage on normal tissues (Figure S29, Supporting Information), indicating the secure and effective therapeutic effect of “nanoenabled energy interrupter.” As expected, more apoptotic cells in HZ@GD group were also observed by TUNEL staining (Figure 5f; and Figure S30a, Supporting Information). To explore the antitumor mechanism of HZ@GD, first, the intratumoral GLUT1 expression was analyzed by immunofluorescence assay. As indicated in Figure 5g; and Figure S30b (Supporting Information), little red fluorescence (GLUT1) existed in tumors after treatment of HZ@GD, while strong red fluorescence could be clearly observed in other groups. This phenomenon proved that “nanoenabled energy interrupter” could effectively down‐regulate the expression of GLUT1. A similar result was also proved by qRT‐PCR (Figure 5h) and western blotting (Figure S31a,b, Supporting Information), respectively. Intratumoral LA and ATP levels were further measured. LA and ATP levels after treatment of HZ@GD were obviously declined by 62.5% and 83.1%, respectively, which was much lower than that of other groups (Figure 5I,j). These results reflected “nanoenabled energy interrupter” embraced the more outstanding ability of energy exhaustion than other formulations, which attributed to the synergistic effect of “Zn^2+^ interference” mediated glycolysis inhibition and Zn^2+^ activating specific GLUT1 depletion.

## 3 Conclusion

To summarize, we successfully designed and fabricated the dual gate‐controlled “nanoenabled energy interrupter” for energy exhaustion induced by preferential “Zn^2+^ interference” in melanoma. According to our results, we proposed that: i) Such a “nanoenabled energy interrupter” not only presents a preferential accumulation tendency to tumor sites due to the active CD44‐targeting mechanisms but also specifically releases Zn^2+^ and DNAzymes controlled by a dual gate (pH and HAase) within tumor cells. ii) “nanoenabled energy interrupter” can induce “Zn^2+^ interference” preferentially in melanoma; iii) based on preferential Zn^2+^ accumulation and high rate of glycolysis in melanoma cells, “nanoenabled energy interrupter” can achieve more pronounced glycolysis inhibition of tumor; iv) combining with energy sources upstream (glucose) cutting off by Zn^2+^‐activated gene silencing, “nano‐enabled energy interrupter” finally resulted in systematic energy exhaustion on melanoma. Our studies thus offer a new tactic to efficiently achieve tumor treatment based on ion homeostasis interference strategy.

## Conflict of Interest

The authors declare no conflict of interest.

## Supporting information

Supporting Information

## Data Availability

Research data are not shared.
