# Supplementary material for: Nano‐enabled Tumor Systematic Energy Exhaustion via Zinc (II) Interference Mediated Glycolysis Inhibition and Specific GLUT1 Depletion
Source: Adv Sci (Weinh). 2021 Dec 16;9(7):2103534. doi: 10.1002/advs.202103534 (PMC8895132; doi:10.1002/advs.202103534)
Supplement: Supplementary file 1 — Supporting Information [file ADVS-9-2103534-s001.pdf]

ADVANCED SCIENCE

Open Access

Supporting Information

for *Adv. Sci.*, DOI: 10.1002/advs.202103534

Nano-enabled tumor systematic energy exhaustion via zinc (II) interference mediated glycolysis inhibition and specific GLUT1 depletion

Sixuan Wu, Kaixiang Zhang, Yan Liang, Yongbin Wei, Jingyi An, Yifei Wang, Jiali Yang, Hongling Zhang, Zhenzhong Zhang, Junjie Liu, Jinjin Shi**

Supporting Information

Nano-enabled tumor systematic energy exhaustion via zinc (II) interference mediated glycolysis inhibition and specific GLUT1 depletion

Sixuan Wu[†,‡], *Kaixiang Zhang*[†,‡], *Yan Liang*[†,‡], *Yongbin Wei*[†,‡], *Jingyi An*[†,‡], *Yifei Wang*[†,‡], *Jiali Yang*[†,‡], *Hongling Zhang*[†,‡], *Zhenzhong Zhang**[,†,‡,§,‖], *Junjie Liu**[,†,‡], *Jinjin Shi**[,†,‡]

[†]School of Pharmaceutical Sciences, Zhengzhou University, Zhengzhou 450001, PR China

[‡]Key Laboratory of Targeting Therapy and Diagnosis for Critical Diseases, Zhengzhou 450001, RP China

[§]Key Laboratory of Advanced Drug Preparation Technologies, Ministry of Education, Zhengzhou 450001, RP China

[‖]State Key Laboratory of Esophageal Cancer Prevention & Treatment, Zhengzhou 450001, RP China

Methods

Materials. Zinc nitrate hexahydrate ($Zn(NO_3)_2 \cdot 6H_2O$), 2,4-dimethylimidazole were purchased from Sigma-Aldrich. Hyaluronic acid (HA, MW< 10 kDa) was purchased from Bloomage Biotechnology Corporation Limited. Zinquin AM (Catalog no. 21261) was purchased from AAT Bioquest. NAD^+ assay kit (Catalog no. BC0310), Glyceraldehyde-3-phosphate dehydrogenase activity kit (Catalog no. BC2215) LA assay kit (Catalog no. BC2230), ATP assay kit (Catalog no. BC0300), Lyso-Tracker Red (Catalog no. L8010-50 μL), Hoechst 33342 (Catalog no. C0030-50 mL) and Dil (Catalog no. D8700-10 mg) were purchased from Solarbio. Nicotinamide phosphoribosyltransferase ELISA Kit (Catalog no. EH0651) was purchased from Wuhan Fine Biotech Co., Ltd. Anti-GLUT1 antibody (Catalog no. 21829-1-AP), Anti-CD44 monoclonal antibody (Catalog no. 60224-1-Ig), Anti-Beta actin monoclonal antibody (Catalog no. 66009-1-Ig), goat anti-mouse IgG secondary antibody conjugated with Cy3 (Catalog no. SA00009-1) and anti-GAPDH antibody (Catalog no. 60004-1-Ig) were purchased from Proteintech. 2-NBDG (Catalog no. B6035) and Cell Counting Kit-8 (CCK-8, Catalog no. K1018) were purchased from APExBIO. Annexin V-FITC/PI (Catalog no. 70-AP101-60) was purchased from Multi sciences. Lipofectamine™ 2000 Transfection Reagent (Catalog no. 11668019) was purchased from Thermo Fisher. IR-783 (Catalog no. 543292) was purchased from Sigma-Aldrich

Characterization. Transmission electron microscopy (TEM) images were obtained by a transmission electron microscope (JEM 1200EX, JEOL, Japan). XRD patterns were gained from X-ray power diffractometer (D8 Advance, Bruker, Germany) in the range of 5-50 degrees (2θ). Size distribution and zeta potential were measured by a Zeta sizer (Nano ZS-90, Malvern, UK). The UV-Vis spectrum was obtained using a UV-Vis spectrometer (UV2700, Shimadzu, Japan). TGA profiles were acquired using a gravimetric analyzer (Q500, TA,USA). The levels of NAD^+, LA, ATP, the activity of GAPDH and cell viability were measured using a microplate reader (Synergy H1, BioTek, USA). The fluorescence images were obtained from a laser scanning confocal microscopy (TCS SP8, Leica, Germany). ICP-MS results were acquired from an inductively coupled plasma mass spectrometer (7500ce, Agilent, USA). In vivo biodistribution was detected using a small animal imaging system (Xtreme, Bruker, Germany). Blood biochemistry analysis was conducted by an Auto biochemical analyzer (Chemray 240, Rayto, China). Blood routine was examined by Auto Hematology Analyzer (BC-2800vet, Mindray, China). Time-lapse imaging was obtained from a LiTone LBS Letsheet machine (Light Innovation technology ltd).

Synthesis of ZIF-8, HZ, Z@GD, HZ@GD and L@GD nanoparticles. ZIF-8 nanoparticles were synthesized following a reported method. 1 mL 160 mM 2-methylimidazole (dissolved in deionized water) was agitated magnetically (300 rpm) for 5 min at room temperature. Then 1 mL 10 mM zinc nitrate hexahydrate (dissolved

in deionized water) was dropwise added. The above solution was incubated for 10 min by mighty magnetic stirring (1200 rpm). After stirring, the precipitate was obtained by centrifugation (12000 rpm, 20 min), and the collected precipitate was washed with deionized water three times. The white powders were dried in a vacuum. The DNAzyme (labeled with AF488) content in the mixture was calibrated by fluorescence intensity and a standard curve of free DNAzyme-AF488. HA-coated ZIF-8 (HZ) was prepared by adding 2 mL 10 mg/mL HA aqueous solution to the above products. The mixture was gently stirred at room temperature for 48 h. Then the samples were washed with deionized water three times to remove free HA. The obtained HZ nanoparticles were dried in a vacuum. Fluorescein (AF488) labeled HZ nanoparticles were synthesized by adding 1 μM AF488 into 1 mL160 mM 2-methylimidazole (dissolved in deionized water), then incubated with 1 mL 10 mM zinc nitrate hexahydrate (dissolved in deionized water) for 5 min at 300rpm. The collected product was modified with HA according to the above procedures. At last, the mixture was collected by centrifugation. Z@GD (ZIF-8@DNAzyme) nanoparticles were synthesized according to other's previous work[1]. Firstly, 2-methylimidazole in deionized water (1 mL, 160 mM) was incubated with DNAzyme (200 mL, 16.5 mM) for 5 min by gentle magnetic stirring. Secondly, 1 mL 10 mM zinc nitrate hexahydrate (dissolved in deionized water) was added dropwise into the above solution, and the mixture was mightily stirred (1200 rpm) for 10 min at room temperature. After stirring, the precipitate was gathered by centrifugation (12000 rpm,

20 min), and the obtained precipitate was washed with deionized water three times. At last, the white powders were dried in a vacuum. To obtain HA-conjugated Z@GD (HZ@GD), the as-prepared Z@GD nanoparticles were dispersed in HA of water solution (2 mL, 10 mg/mL). The mixture was gently stirred at room temperature for 48 h. Then, the samples were washed with deionized water three times to remove free HA. The obtained HZ@GD nanoparticles were dried in a vacuum. In addition, the encapsulation efficiency was calculated by fluorescence spectrophotometer collecting the emission at 488 nm (AF488 emission maximum) for preparing a pre-determined calibration curve, and measuring the DNAzyme concentrations in the precursor solution and the supernatant of the obtained nanoparticles. L@GD nanoparticles were prepared by incubating 500 nM DNAzymes with 5 μL Lipo 2000 (2500 ng) in FBS for 30 min.

In vitro Zn^{2+} Release from HZ@GD. HZ@GD nanoparticle (100 μg/mL) was sufficiently dissolved into PBS buffer (pH 7.4, pH 6.8, pH 5.0) with or without HAase (0.2 mg/mL) for different time points, respectively. Subsequently, the mixture was centrifuged at 12000 rpm for 20 min, the supernatant was collected for detecting Zn^{2+} concentrations by inductively coupled plasma mass spectrometry (ICP-MS). Meanwhile, 100 μg/mL 1 mL HZ at different pHs (pH 7.4, 6.8, and 5.0) was utilized to examine the change of UV absorbance from 200 nm to 800 nm by a UV-Vis spectrometer (UV2700, Shimadzu, Japan).

In vitro DNAzyme release from HZ@GD. HZ@GD (DNA was labeled with AF488) (1 mL, 200 μg/mL) was loaded into a dialysis tube (MWCO, 3 kDa) against 50 mL of PBS buffer (pH 7.4 and 5.0) with or without HAase (0.2 mg/mL) at 37 ^{o}C, 150 rpm under lucifuge environment. At timed intervals, 0.5 mL of the buffer solution was taken out and acidized with the PBS buffer (0.5 mL). The fluorescence intensity was measured by a microplate reader (Ex: 488, Em: 525) (Synergy H1, BioTek, America). The DNA concentration was calculated by fluorescence intensity according to a standard curve of free DNAzyme-AF488.

Disintegration of HZ@GD in vitro. HZ@GD nanoparticle (100 μg/mL) was sufficiently dissolved into PBS buffer (pH 7.4, pH 6.8, pH 5.0) with or without HAase (0.2 mg/mL) for 5 h, respectively. Subsequently, the mixture was centrifuged at 12000 rpm for 20 min, the precipitation was collected for TEM characterization. Transmission electron microscopy (TEM) images were obtained by a transmission electron microscope (JEM 1200EX, JEOL, Japan).

Experiment of fluorescence-resonance energy transfer (FRET). Firstly, 50 μL DNAzyme solution (500 nM) was mixed with 50 μL GLUT1 substrate solution (500 nM) for 1 h. Then, 50 μL $ZnCl_2$ solution was separately added to the above solution and the final Zn^{2+} concentration was up to 0, 1, 5, 10 μM. Immediately, the mixture (50 μL) of fluorescent chain (250 nM) and quenching chain (250 nM) was added to the above solution. Lastly, a real-time fluorescence intensity was minitored by a microplate reader (Ex: 488, Em: 525).

Experiment of DNAzyme and HZ@GD cleavage in vitro. The experiment of DNAzyme cleavage in vitro was carried out: DNAzyme (500 nM) was incubated with an equal amount of GLUT1 substrate (500 nM) (Table S1) at room temperature for 5 h in Tris-HCl buffer (pH = 6.8) containing different concentrations of $ZnCl_2$ (1 μM, 5 μM, 10 μM), respectively. After incubation, the cleavage efficiency was verified by polyacrylamide gel electrophoresis. Different concentrations of HZ@GD were incubated in PBS buffer (pH 7.4, 6.8 and 5.0) containing 0.2 mg/mL HAase for 5 h. After incubation, the solution pH was adjusted to pH 7.4, then GLUT1 substrate (500 nM) was added into the above solution. After the mixture was incubated for 5 h, the cleavage efficiency was verified by polyacrylamide gel electrophoresis.

Cell culture. B16-F10 melanoma cell line was kindly provided by the China Center for Type Culture Collection at Wuhan University (Wuhan, Hubei, China). The HL-7702 cell line and PIG1 cell line were purchased from the Procell Life Science & Technology Company (Wuhan, Hubei, China). B16-F10 cells, HL-7702 and PIG1 cells were maintained RMPI 1640 (Solarbio) supplemented with 10% fetal bovine serum (Gibco, Invitrogen) and 1% penicillin-streptomycin solution (100 U/mL, Invitrogen) at 37°C under 5% CO2. All experiments were conducted on cells in the exponential growth phase.

Cellular uptake assay. B16-F10 and PIG1 cells were seeded at a 35 mm confocal dish at 1×10^5 per dish and cultured for 12 h. After that, cells were washed with PBS three times and incubated with HZ@GD (labeled with AF488) (200 μg/mL)

for 1 and 4 h. Meanwhile, to verify the CD44-mediated targeting uptake mechanism, anti-CD44 antibody firstly were incubated with B16-F10 and PIG1 cells for 2h, then cells were washed with PBS three times and incubated with HZ@GD (labeled with AF488) (200 μg/mL) for 4 h. After incubation, cells were washed with PBS three times and stained with 1 mL Hoechst 33342 for 10 min. After staining, images were acquired using a Leica laser scanning confocal microscope (TCS SP8, Leica, Germany). Lastly, the cells were collected by trypsin digestion. The fluorescence intensity of collected cells was detected by a flow cytometer (BD Accuri®C6, American).

Assessment of cellular uptake efficiency. B16-F10 and PIG1 cells were seeded at 35 mm confocal dish at 1×10^5 per dish and cultured for 12 h. After that, cells were washed with PBS for three times and incubated with HZ@GD (labeled with AF488) (50 μg/mL) for 4 h. After incubation, cells were collected by trypsin digestion and fluorescence intensity was detected by a flow cytometer (BD Accuri®C6, American). To set the same uptake amount, B16-F10 cells and PIG1 cells were exposed to 50 and 80 μg/mL HZ-FITC, respectively. After they were incubated for 1 h, PBS was used to wash B16-F10 cells and PIG1 cells for three times. Then, the cells were digested by 0.25% trypsin and were obtained by centrifugation. The cell pellet was resuspended in PBS for flow cytometry analysis. B16-F10 cells were cultured according to the above process. Then cells were washed with PBS three times and incubated with HZ@GD-AF488 (200 μg nanoparticles containing 8 μg DNAzyme) and L@GD-AF488 (8 μg

DNAzyme) for 4 h. After incubation, cells were collected by trypsin digestion and fluorescence intensity was detected by a flow cytometer (BD Accuri®C6, American).

Lysosome escape assay. Cells were seeded at a 35 mm confocal dish at 1×10^5 per dish and cultured for 12 h. After that, cells were washed with PBS for three times and incubated with HZ@GD (labeled with AF488) (200 μg/mL) for 0.5, 1 and 2 h. After incubation, cells were washed with PBS for three-time and stained with 1 mL Hoechst 33342 for 10 min. Then cells were washed with PBS for three times and incubated with Lyso-Tracker Red (75 nM) for 5 min. After staining, images were acquired and the person correlation coefficients were analyzed by a Leica laser scanning confocal microscope (TCS SP8, Leica, Germany). For Bio-TEM assay, B16-F10 cells grown at logarithmic phase were incubated with 50 μg/mL HZ for 24 h. The untreated cells were used as control cells. After incubation, cells were digested and centrifuged. The obtained cell pellets were fixed with 2.5% glutaric acid and 1% osmium acid for 2h, respectively. Then the cells were washed with PBS for three times. The clean samples were dehydrated with gradient concentrations of ethanol solution (including 30%, 50%, 70%, 80%, 90% and 95%) for 15min, respectively, and then the cells were treated with 100% ethanol for 20min. At last, the cells were incubated with pure acetone for 20min.The sample was treated with the mixture of embedding agent and acetone (V/V=1/1) for 1h and treated with a mixture of embedding agent and acetone (V/V=3/1) for 3h. The treated samples were embedded with pure embedding agent overnight; The permeated sample is embedded and heated

overnight at 70℃ to obtain the embedded sample. The samples were sliced in LEICA EM UC7 ultra-thin slicer to obtain 70-90nm slices, which were stained with lead citrate solution and 50% ethanol saturated solution of dioxane acetate for 5-10min respectively, and then dried for observation under transmission electron microscope.

Intracellular free Zn^{2+} accumulation by HZ. Intracellular free Zn^{2+} accumulation by HZ. Cells were seeded at 35 mm confocal dish at 1×10^5 per dish and cultured for 12 h. After that, cells were washed with PBS for three times and incubated with HZ (50 μg/mL) for 0 h, 1 h, and 2 h, respectively. For TPEN group, B16-F10 cells were firstly incubated with 50 μg/mL HZ for 2 h and then treated with 1 μM TPEN for 0.5 h, respectively. After incubation, cells were washed with PBS for three times. Subsequently, the FluoZin-3 probe (50 μM, 30 min) and DiI probe (200 nM, 10 min) were incubated with cells for indicating intracellular free Zn^{2+} accumulation and staining cell membranes, respectively. Lastly, a Leica laser scanning confocal microscope was utilized for characterizing fluorescence intensity. Flow cytometer assays are performed following the cell operations described above, then 50 μM FluoZin-3 probe was incubated with cells for 30 min. For time-lapse imaging, B16-F10 cells firstly were stained with 50 μM FluoZin-3 probe for 30 min. The stained cells are immersed in 50 μg/mL HZ solution, and then the change of blue fluorescence is observed in real-time using a LiTone LBS Letsheet machine (Light Innovation technology ltd).

Quantitative analysis of intracellular levels of free Zn^{2+} from HZ. Cells were seeded at a 35 mm confocal dish at 1×10^5 per dish and cultured for 12 h. After that, cells were washed with PBS for three times and incubated with HZ (50 μg/mL) for 0 h, 1 h, and 2 h. After incubation, cells were washed with PBS for three times and collected by trypsin digestion. The collected cells were resolved by nitric acid and hydrogen peroxide (30%, 3 mL). The mixture was added with deionized water to 10 mL. The final solutions were used to measure the concentration of Zn^{2+} by ICP-MS.

Measurement of NAD^+, LA ATP levels and the activity of GAPDH. NAD^+ ATP levels and the enzymatic activity of GAPDH were measured: Cells were seeded at 6-well plates at 1×10^5 per well and cultured for 12 h, and then incubated with the different concentrations of HZ (10 μg/mL, 30 μg/mL, 50 μg/mL) for 12 h. For TPEN group, B16-F10 cells were treated with 50 μg/mL HZ and 1 μM TPEN for 12 h. After incubation, cells were absolutely collected by trypsin digestion and centrifugation (1000 rpm, 5 min). The obtained precipitate of cells was used to measure cellular NAD^+, ATP levels and enzymatic activity according to the instruction of NAD^+ ATP and the enzymatic activity of GAPDH testing kits, respectively. LA levels were measured: Cells were cultured and treated with HZ according to the above process. After treating, the supernatant of cells was gained for detecting secretory LA content according to the instruction of LA testing kits.

RNA-seq for transcriptome analysis. B16-F10 cells were seeded at 6-well plates at 1×10^5 per well and cultured for 12 h, and then were incubated with 50 μg/mL

of HZ for 12 h. Untreated B16-F10 cells were used as control cells. Each group had three parallel replicates. After HZ incubation, cells were washed, digested and centrifugally collected for transcriptome analysis. Total RNA extraction, RNA sequencing and bioinformatic data collection were performed by Saicheng Biotechnology Co., LTD (Guangzhou, China).

GLUT1 measurement via qRT-PCR and western blot. The qRT-PCR assays were carried out as follows: cells were seeded at 6-well plates at 1×10^5 per well and cultured for 12 h, and then were incubated with 50 μg/mL of different nanoparticles (L@GD, HZ, HZ@RD, HZ@GD) for 12 h, and 2 μg DNAzyme in the nanoparticles was applied to cleave GLUT1 mRNA. After being washed with PBS, cells were incubated with fresh complete medium for 12 h. After incubation, precipitate of cells was completely collected by trypsin digestion and centrifugation (1000 rpm, 5 min). Then mRNA was isolated with a Trizol Reagent kit. One microgramme of RNA sample was utilized to obtain cDNA. One microliter of cDNA sample and specific primers (Table S2) were used to amplify GLUT1 cDNA according to the manufacturer's parameters (Custom gene qRT-PCR Quantitation Kit) on a Real-Time PCR machine. The western blot assays were carried out as follows: cells were treated according to the above same method. The cells were collected and washed twice with PBS. Cell precipitations were lysed for 1 h at 4 ^{o}C. The protein content was measured by Bradford assay. Western blot analysis was conducted using standard method and the β-actin was used as internal references.

CD44 Measurement via western blot. The western blot assays were carried out as follows: B16-F10 cells, PIG1 cells and HL-7702 cells were seeded at 6-well plates at 1×10^5 per well and cultured for 12 h. The cells were collected and washed twice with PBS. Cell precipitations were lysed for 1 h at 4 ^{o}C, and then centrifugated for 12000 g, 10 minutes. The protein content was measured by Bradford assay. Western blot analysis was conducted using standard method.

Measurement of Glucose uptake efficiency. B16-F10 cells were treated with 50 μg/mL of different nanoparticles (L@GD, HZ, HZ@RD, HZ@GD) for 12 h, and then cells were incubated with a fresh complete medium for 12h. After incubation, cells were washed with PBS for three times. Subsequently, the treated cell was incubated with fluorescent glucose analog (2-NBDG, 100 μM) for 20 min. After incubation, the cells were washed with PBS and then photographed by a Leica laser scanning confocal microscope (TCS SP8, Leica, Germany). At last, the cells were collected by trypsin digestion. The fluorescence intensity of collected cells was detected by a flow cytometer (BD Accuri®C6, American).

Cytotoxicity assay. The in vitro cytotoxicity assay of HZ and HZ@GD was conducted with CCK8 assay following the literature procedure. B16-F10 cells were seeded in 96-well plates at 1×10^4 per well and cultured in 100 μL fresh complete medium for 12 h. Another complete medium containing HZ or HZ@GD (5, 10, 30, 50, 100 and 200 μg/mL) were added, respectively. After incubating for 24 h, 10 μL CCK8 was added into the medium and kept for another 2 h. The optical density (OD) was

measured at 450 nm by a microplate reader (Synergy H1, American). Cell viability of different nanoparticles was calculated by the following formula: cell viability (%) = $(OD_{sample} – OD_{blank} / OD_{control} – OD_{blank}) \times 100\%$.

Apoptosis assay. B16-F10 cells were seeded at 6-well plates at 1×10^5 per well and cultured for 12 h. After 12 h culture, cells was incubated with 1 mL medium containing different nanoformulations (L@GD, HZ. HZ@RD and HZ@GD) for 24 h. The doasge of HZ, HZ@RD and HZ@GD in medium are all 50 μg/mL. The concentration of DNAzyme in L@GD, HZ@RD and HZ@GD is 13.5Mm, respectively. Each group had three parallel replicates. After incubating for 24, cells were collected by digestion and centrifugation. The obatined cells was stainned with Annexin V-FITC and PI based on the standard apoptotic detection prodecures. Lastly, the fluorescence intensity of cells was analyzed by a flow cytometer (BD Accuri®C6, American).

Animal experiments. Four- to six-week-age BALB/C or C57BL/6 mice (18-22 g) were feed at the condition of 25°C and 55% of humidity in Experimental Animal Center of Zhengzhou University. All the animal experiments were performed in accord with the guidelines of the Regional Ethics Committee for Animal Experiments and the Care Regulations approved by the Institutional Animal Care and Use Committee of Zhengzhou University. The license number of experimental animal is SCXK (Beijing) 2017-0005.

Hemolysis assay. Hemolysis assay was conducted according to the reported method. Fresh blood cells of mice were isolated in vacutainers containing EDTA. The samples were centrifuged at 2000 rpm for 5 min and washed with saline (0.9% NaCl) for three times. At last, blood cells were suspended in 1 mL saline to prepare the stock solution. The suspensions were 1:10 diluted by saline and kept on ice and ready for the hemolysis assay. HZ@GD was dissolved in 800 μL of saline at an HZ@GD dose of 50, 100, 500 and 1000 μg/mL, respectively. The obtained RBC suspensions (200 μL) were added to the solution. These samples were incubated in an incubator at 37℃ for 12 h. The cells were then centrifuged at 10000 rpm for 10 min. The absorbance of the supernatant was measured at 541 nm. The absorbance of the supernatant of RBC in saline and deionized water was used as 0% hemolysis and 100% hemolysis controls, respectively. The cell hemolysis rate was calculated by Hemolysis(%)=(Abs-Abs_0)/(Abs_{100}-Abs_0) ×100 %, Where Abs, Abs_{100}, and Abs_0 are the absorbance scales of the sample, the 100% and 0% hemolysis solution, respectively.

Serum biochemistry assay. The B16-F10 tumor-bearing mice were divided into five groups: saline, L@GD, HZ, HZ@RD, HZ@GD. The different formulations (DNAzyme: 100 μg/mL) were intravenously injected. The blood of mice was collected after injection 24 h. The samples were utilized to examine those parameters of blood biochemistry and blood routine by Auto biochemical analyzer (Chemray 240, Rayto, Shenzhen) and Auto Hematology Analyzer (BC-2800vet, Mindray, China), respectively.

In vivo biodistribution. The distribution of formulations was imaged in vivo: ZIF-8@IR783 was synthesized by one-pot synthesis: In brief, IR783 (200 μL, 200 μg/mL) and 2-methylimidazole in deionized water (1 mL, 160 mM) were completely mixed by agitation (300 rpm, 5 min), zinc nitrate hexahydrate in deionized water (1 mL, 10 mM) was added into the mixture under mighty stirring for 10 min at room temperature. After the interaction, ZIF-8@IR783 was obtained by centrifugation and washed thrice using deionized water. HA/ZIF-8@IR783 was synthesized by incubating HA with ZIF-8@IR783 for 48h at room temperature. The tumor-bearing mice were randomly divided into three groups and intravenously injected free IR783, ZIF-8@IR783 and HA/ZIF-8@IR783 (the IR783 concentration of each group: 8 μg/mL). The fluorescence and X-Ray images were collected at the predetermined time intervals post-injection (1, 2, 6, 12, 24 and 48 h) by a small animal imaging system (Xtreme, Bruker, Germany). Then mice were executed and the tumor and major tissues (heart, liver, spleen, lung, and kidney) were gained and photographed. The metabolism of HZ@GD in different tissues was measured as follows: The B16-F10 tumor-bearing mice were divided into three groups and were intravenously injected HZ@GD (1.6 mg/mL, 200 μL), then the mice were sacrificed at the predetermined time intervals post-injection (0 h, 24 h and 48 h). The collected various organs were washed with saline and weighted. Subsequently, all the organs were resolved by nitric acid and hydrogen peroxide (30%, 3 mL) at 100 ^{o}C. The final solutions were used to measure the concentration of Zn^{2+} by ICP-MS. The assays of

Zn^{2+} accumulation of HZ@GD in tumors were carried out: The B16-F10 tumor-bearing mice were divided into two groups and intravenously injected saline and HZ@GD (1.6 mg/mL, 200 μL), respectively. The tumor was collected after injection 24 h for free Zn^{2+} staining according to the following procedure: the collected tumors were fixed immediately in 10% paraformaldehyde solution, followed by standard dehydration and paraffin embedding. The embedded tumors were then sectioned into 4 μm slices and Zinquin stained. Briefly, the slides were stained with zinquin probe (50 μM) for 1 min and were washed with PBS for 1 min. The final slides were visualized under a Leica laser scanning confocal microscope (TCS SP8, Leica, Germany).

In vivo anti-tumor efficiency. Briefly, the B16-F10 tumor-bearing C57BL/6 mice were divided randomly into five groups: saline, L@GD, HZ, HZ@RD, HZ@GD. The different formulations (DNAzymes: 100 μg/mL) were administrated through tail vein injection every other day for 2 weeks, respectively. Body weights and tumor sizes were monitored every other day after the corresponding treatments. The tumor volume was calculated by the equation: Tumor volume = $(width^2 \times length)/2$. Relative tumor volume was the corresponding changes relative to the initial value measured before treatment. All mice were photographed and euthanized at two weeks postinjection, and the tumors were taken out and photographed. The collected organs and tumors were fixed immediately in 10% paraformaldehyde solution, followed by standard dehydration and paraffin embedding. The embedded tissues were then

sectioned into 4 μm slices and then subjected to standard H&E and Tunnel staining for histological analysis.

In vivo efficiency of energy exhaustion. Fluorescent immunohistochemistry of GLUT1 in tumor: the 4 μm-thickness tumor slides were blocked with PBS containing 5% normal goat serum for 1 h at RT. Afterwards, these slides were incubated with anti-GLUT1 primary antibody (Proteintech, Catalog no. 21829-1-AP) in PBS containing 1% goat serum overnight at 4 ℃ to stain GLUT1 protein. Primary antibody binding was visualized using goat anti-mouse IgG secondary antibody conjugated with Cy3 (Proteintech, Catalog no. SA00009-1). Cell nuclei were counterstained with DAPI for 10 min before slides were mounted and subject to fluorescent microscopy (TCS SP8, Leica, Germany). Measurement of LA and ATP levels in tumor: tumor-bearing C57BL/6 mice after treatment with formulations were executed. Exfoliated tumor tissues were randomly cut out three sections and the weight of every section was up to 0.1 g. The LA and ATP levels of these samples were measured according to the instruction of LA and ATP testing kits, respectively.

Statistical Analysis. All data were shown as mean ± s.d. Statistical analysis was conducted via a Student's test using GraphPad Prism 8.01. *P*-values were determined by a two-tailed unpaired t-test. *P<0.05, **P<0.01, ***P<0.001, ****P<0.0001.

Table S1. The sequences of GLUT1 DNAzyme and substrate.

Name	Sequence(5'~3')

**GLUT1 DNAzyme (Mus musculus)**	**ACCAGGGCTCCGAGCCGGTCGAAACTTCAAAGA**
**Substrate (Mus musculus)**	**TCTTTGAAGT/rA/GGCCCTGGT** [a)]
**Random sequence DNAzyme**	**ACCAGGGCTAAAAAAAAAAAAAAACTTCAAAGA**
**GLUT1 DNAzyme (Homo sapiens)**	**AGCGAGGCTCCGAGCCGGTCGAAATGAGGTGCA**
**Substrate (Homo sapiens)**	**TGCACCTCAT/rA/GGCCTCGCT**

a) is a deoxyribose adenine.

Table S2. Primers used to amplify GLUT1 cDNA.

Name	Sequence(5'~3')
**MusGLUT1 F primer**	**TGGCTTTGTGGCCTTCTTTGA**
**MusGLUT1 R primer**	**GAGAAGCCAGCCACAGCAATA**

Reference

[1] H. Wang, Y. Chen, H. Wang, X. Liu, X. Zhou, F. Wang, Angewandte Chemie 2019, **58**, 7380.

The schematic diagram of GLUT1 DNAzyme to shear GLUT1 substrate

The DNAzyme make up of deoxynucleotide sequences, which contains a 15 highly conserved deoxyribonucleotides that called as catalytic domain and can cleave the scissile dinucleotide junction, flanked by two substrate-recognition sequences that are complementary to target and bind RNA substrate specifically.

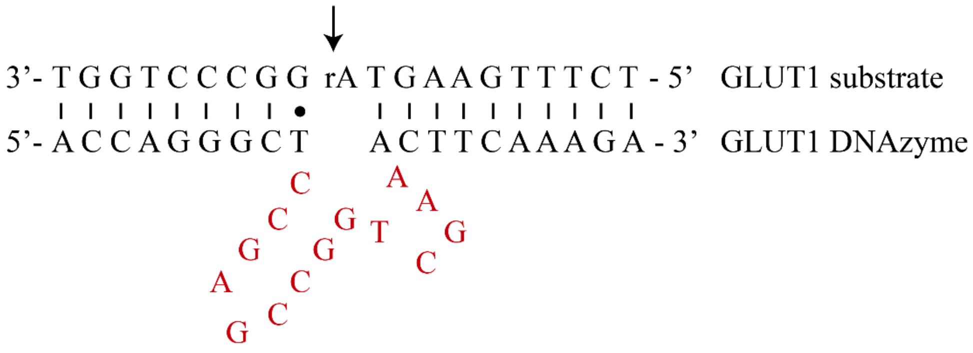

Figure S1. The secondary structure of GLUT1 DNAzyme-GLUT1 substrate complex. The arrow indicates the cleavage site of GLUT1 substrate. rA represents ribonucleic adenine, which is more liable to cleavage than deoxyribonucleotides. In GLUT1 DNAzyme sequence, the red sequence indicates the catalytic domain that can be specifically activated with Zn^{2+}, the black sequence indicates two substrate-recognition sequences.

TEM characterization of ZIF-8, HZ and Z@GD

ZIF-8, HZ and Z@GD were characterized by transmission electron microscopy (TEM), and they all exhibited uniform spherical-like morphology, indicating the successful synthesis of ZIF-8, HZ and Z@GD.

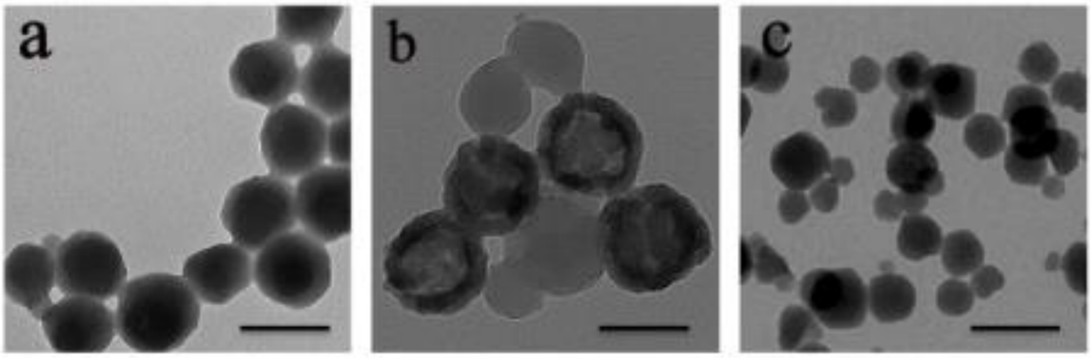

Figure S2. Representative TEM images of (a) ZIF-8, (b) HZ, (c) Z@GD. Scale bar: 100 nm.

Encapsulation efficiency analysis

The high loading efficiency of DNAzymes into HZ NPs was found and the loading efficiency of DNAzymes was determined to be ~81.5%.

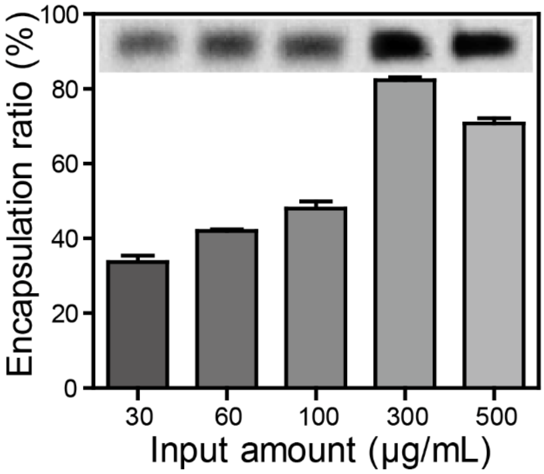

Figure S3. Analysis of encapsulation efficiency under different DNAzyme input amounts (n=3).

TGA analysis of ZIF-8, HZ and HZ@GD

The thermal gravimetric analysis (TGA) was carried out on ZIF-8, HZ and HZ@GD to demonstrate the modification of HA and encapsulation of DNAzyme. The deeper drop was observed with the modification of HA and encapsulation of DNAzyme, respectively, implying that the modified HA and encapsulated DNAzyme affected the thermostability of ZIF-8.

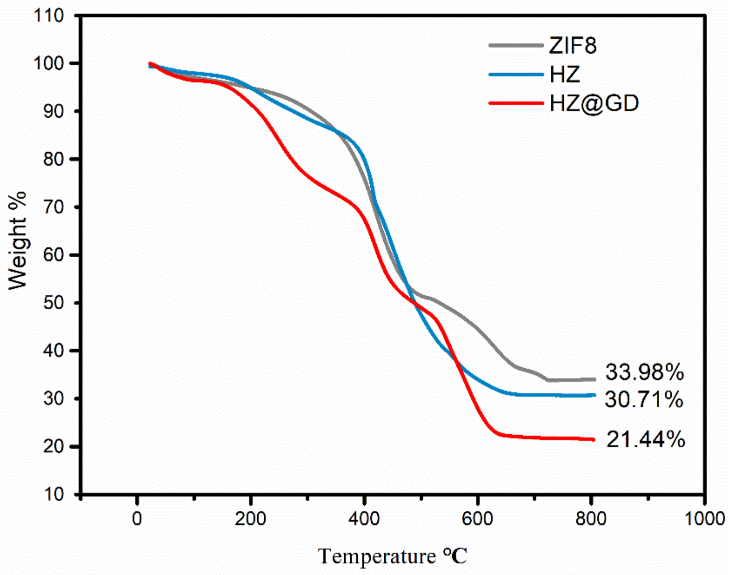

Figure S4. TGA curves of ZIF-8, HZ, and HZ@GD, respectively. Test temperature ranges 0 ℃ from 800 ℃. The heating rate is set to 20 ℃/min.

The size changes of HZ@GD in physiological fluids

To further investigate the stability of HZ@GD nanoparticles, we measured the size distribution of the preparation dispersed in PBS, saline, or cell medium for one week. The preparation can be kept stably in these fluids for at least seven days, and the average hydrodynamic diameter measured is about 80 nm.

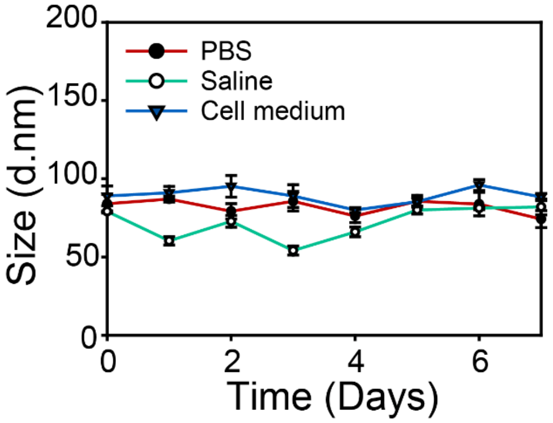

Figure S5. The size changes of HZ@GD in PBS, saline or cell medium (n=3).

Powder X-ray diffraction (PXRD) analysis

To analysis the crystal structure of HZ@GD, the assay of powder X-ray diffraction (PXRD) was conducted. As shown in Figure S6, the uniform spherical-like HZ@GD nanostructures retained a high crystallinity.

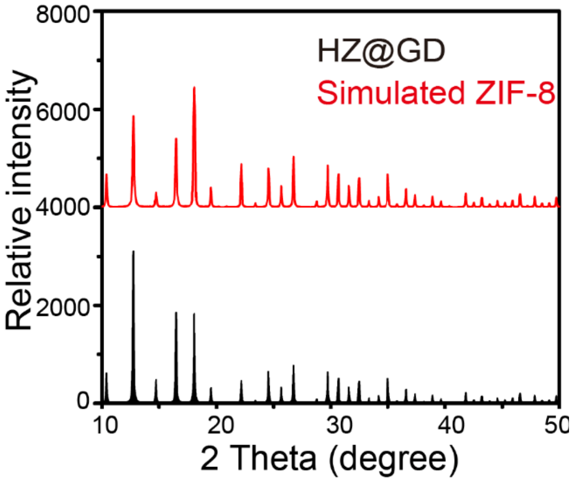

Figure S6. X-ray diffraction (XRD) analysis of HZ@GD

UV-vis analysis of HZ at different pHs

UV-vis analysis was carried out for evaluating the pH-responsive ability of HZ. The decline of absorbance was observed from pH 7.2 to pH 4.5, demonstrating the MOF-based skeleton collapse of HZ@GD under acidic PBS.

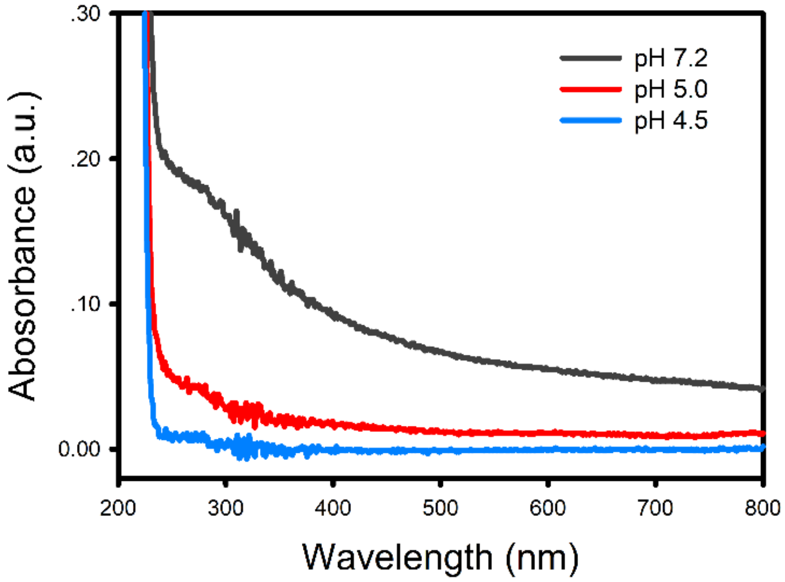

Figure S7. UV-Vis spectrums of HZ at different pHs (7.4, 5.0, 4.5)

Zn^{2+} and DNAzymes released from Z@GD

The characteristics of Zn^{2+} and DNAzymes from Z@GD were characterized by ICP-MS assay and fluorescence analysis, respectively. As reflected in Figure S8, both Zn^{2+} and DNAzymes were efficiently released from Z@GD under pH 6.8 or 5.5 PBS and the release rates were faster than that under a physiological environment (pH = 7.4). While the addition of HAase in these solutions had a negligible effect on these drugs release from Z@GD.

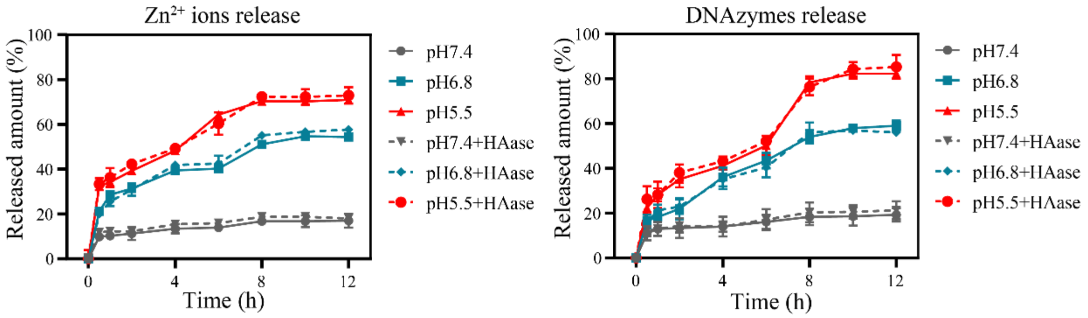

Figure S8. In vitro Zn^{2+} and DNAzymes release curves of Z@GD in PBS at pH 7.4, pH 6.8, and pH 5.5 with or without HAase (0.2 mg/mL) (n=3). All data are shown as the Mean ± SD from three independent experiments.

Enhanced tumor-targeting capability of HZ@GD

The tumor-targeting ability of HZ@GD (labeled with AF488) was analyzed by CLSM and flow cytometer, respectively. B16-F10 cells (melanoma cells) and PIG1 cells (melanocytes) were used as model cells. With the increasing incubation time, more obviously green fluorescence of AF488 inside B16-F10 cells treated with HZ@GD was markedly enhanced than that inside PIG1 cells, indicating that HZ@GD is internalized more efficiently into tumor cells. Furthermore, an obvious decrease of uptake amount of HZ@GD was found in B16-F10 cells pretreated with anti-CD44 antibody than that in PIG1 cells, reversely suggesting that CD44-mediated actively targeting mechanism facilitates the uptake of HZ@GD in tumor cells.

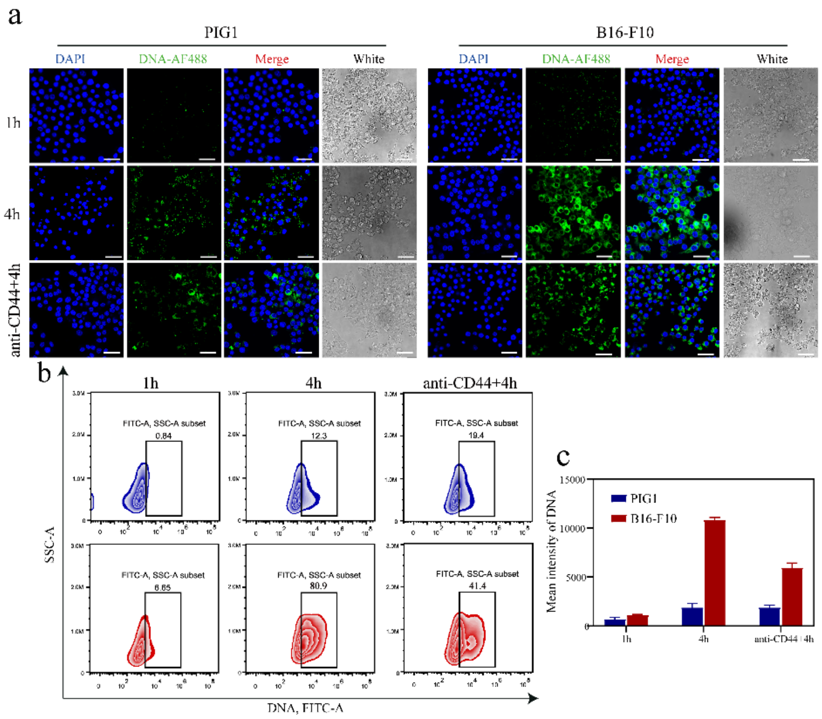

Figure S9. a, Representative CLSM images of B16-F10 cells and PIG1 cells incubated with HZ@GD-AF488, respectively. Scale bar: 25 µm. b, c, Flow cytometry analysis (b) and quantitative data (c, n=3) in B16-F10 cells and PIG1 cells incubated with HZ@GD-AF488, respectively. All data are shown as the Mean ± SD from three independent experiments.

HZ results in significant Zn^{2+} interference in vitro

To assess the intracellular Zn^{2+} accumulation ability of HZ, ICP-MS was conducted for quantitatively analyzing the generation of intracellular Zn^{2+} in B16-F10 and PIG1 cells. The level of Zn^{2+} in B16-F10 cells cultured with HZ was dramatically increased from 0.71 μg/10^6 cells to 4.20 μg/10^6 cells and 7.80 μg/10^6 cells, respectively. While the level of Zn^{2+} exhibited a modest increase in PIG1 cells incubated with HZ from 2.10 μg/10^6 cells to 3.80 μg/10^6 cells and 5.30 μg/10^6 cells, respectively. The result revealed that "nano-enabled energy interrupter" triggers more pronounced fluctuations of Zn^{2+} levels in B16-F10 cells than that in PIG1 cells owing to the inherent lower Zn^{2+} levels in B16-F10 cells, which make melanoma more vulnerable to accumulation of Zn^{2+}.

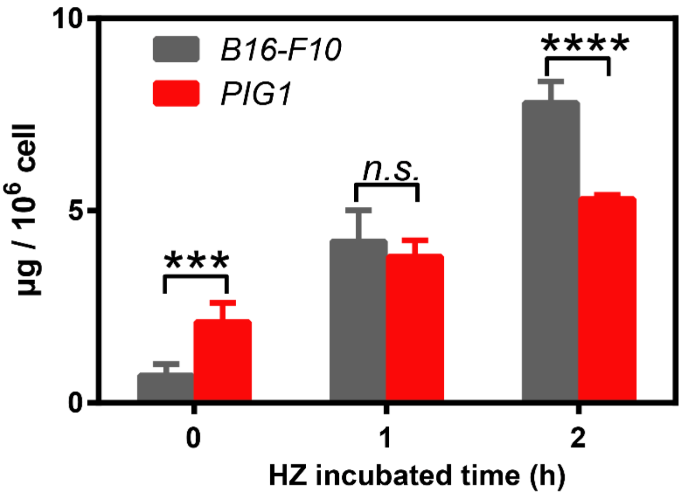

Figure S10. The effect of HZ treatment on Zn^{2+} accumulation of B16-F10 and PIG1 cells. (n=3). All data are shown as the Mean ± SD from three independent experiments. *P*-values were determined by a two-tailed unpaired t-test. **P*<0.05, ***P*<0.01, ****P*<0.001, *****P*<0.0001

Flow cytometry analyze intracellular Zn^{2+} accumulation

Taking advantage of a free Zn^{2+} indicator (FluoZin-3 probe), intracellular free Zn^{2+} levels were tested in B16-F10 cells and PIG1 cells via flow cytometer. As expected, the B16-F10 cells co-incubated with HZ showed a higher mean fluorescence intensity in comparison with the PIG1 cells, indicating a higher intracellular accumulation of free Zn^{2+} in tumor cells. In addition, TPEN, a specific Zn^{2+} chelator, attenuated the mean fluorescence intensity of blue fluorescence induced by HZ, which confirmed that the increased fluorescence intensity is caused by the accumulation of free Zn^{2+}.

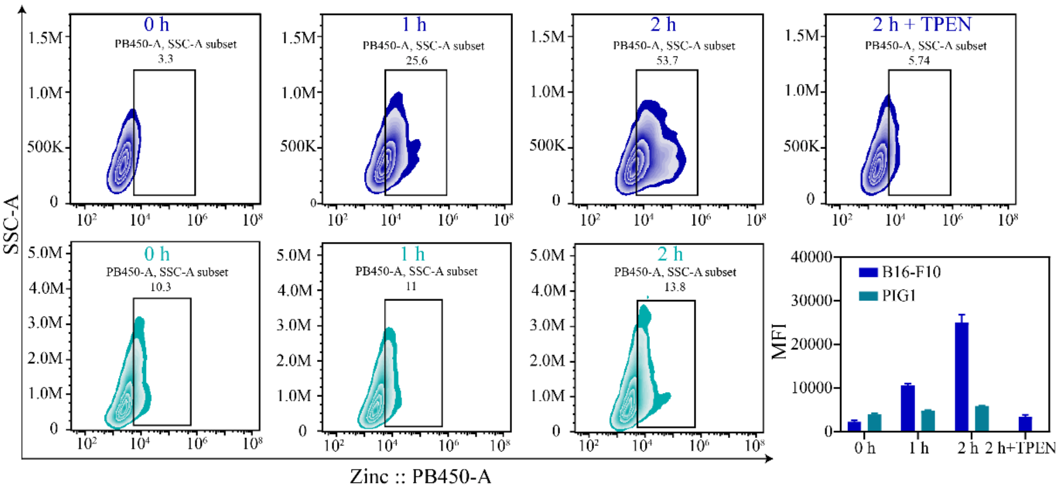

Figure S11. Flow cytometry analysis of intracellular free Zn^{2+} production after treatment with HZ on B16-F10 pre-treated without or with TPEN and PIG1 cells for 0 h, 1 h and 2 h, respectively (n=3). All data are shown as the Mean ± SD from three independent experiments.

Time-lapsing imaging of Zn^{2+} accumulation in a single cell

To further demonstrate Zn^{2+} accumulation into the cytoplasm, the time-lapsing imaging of lysosomal escape in a single cell was obtained by a Letsheet machine. B16-F10 cells exposure with HZ resulted in a gradual increase in blue fluorescence along with the time increment, which means more Zn^{2+} were accumulated in tumor cells.

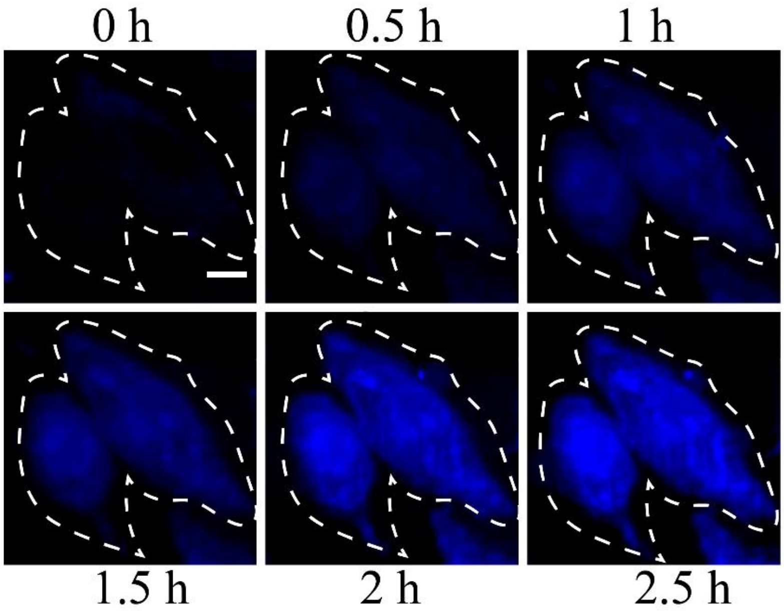

Figure S12. Time-lapse imaging of Zn^{2+} accumulation in B16-F10 cells after treatment with HZ. Scale bar: 5 μm. Blue fluorescence reflected the free Zn^{2+} amount. The white dotted box was the outline of the B16-F10 cell.

Expression difference of CD44 between tumor cells and normal cells

The expression difference of CD44 level on different types of cells (B16-F10 cells, PIG1 cells and HL-7702 cells) was verified by western blotting assay. As expected, compared with PIG1 and GL-7702 cells, there is a more significant expression level of CD44 protein on B16-F10 cells, which provides excellent guarantee of HA-mediated tumor targeting capability by HZ nanoparticles.

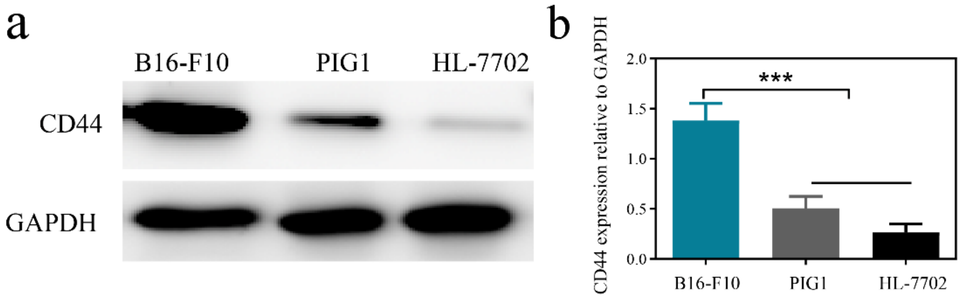

Figure S13. a,b, Western blot assay (a) and Semi-quantitative analysis (b, n=3) of CD44 protein in B16-F10 cells, PIG1 cells and HL-7702 cells, respectively. All data are shown as the Mean ± SD from three independent experiments. *P*-values were determined by a two-tailed unpaired t-test. **P*<0.05, ***P*<0.01, ****P*<0.001, *****P*<0.0001.

Investigation of intracellular uptake efficiency of HZ@GD-AF488 on B16-F10 cells and PIG1 cells

The result of flow cytometer showed that there is the same degree of HZ uptake between B16-F10 cells and PIG1 cells, when B16-F10 cells were incubated with 50 μg/mL HZ-FITC for 1 h, as well as PIG1 cells were incubated with 80 μg/mL HZ-FITC for 1 h.

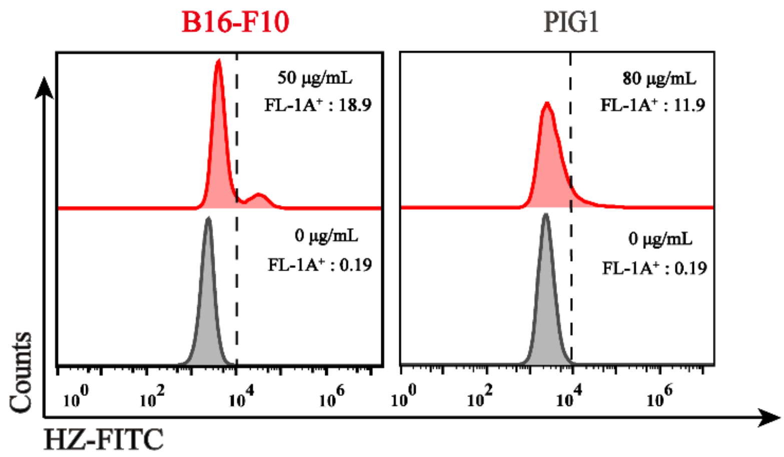

Figure S14. Flow cytometry analysis of B16-F10 cells and PIG1 cells incubated with HZ@GD-AF488, respectively.

Flow cytometer analysis of Zn^{2+} fluctuation in B16-F10 and PIG1 cells

Intracellular Zn^{2+} level fluctuation in B16-F10 and PIG1 cells after treatment with HZ by Flow cytometer. The content of intracellular free Zn^{2+} in B16-F10 and PIG1 cells increased with the prolongation of incubation time of HZ. While, compared with the HZ treated PIG1 cells, the intracellular free Zn^{2+} fluctuation was significantly stronger in the case of HZ treated B16-F10 cells, indicating HZ resulted in more obvious Zn^{2+} accumulation in malignant melanoma cells. Interestingly, there is a significant difference in the basic Zn^{2+} levels between B16-F10 cells and PIG1cells, the original Zn^{2+} content of B16-F10 cells significantly lower than that of PIG1 cells, which is an underlying mechanism that malignant melanoma cells are more susceptible to exogenous zinc disturbance. More importantly, TPEN, a specific Zn^{2+} chelator, attenuated the blue fluorescence induced by HZ, which confirmed that the increased blue fluorescence is caused by Zn^{2}.

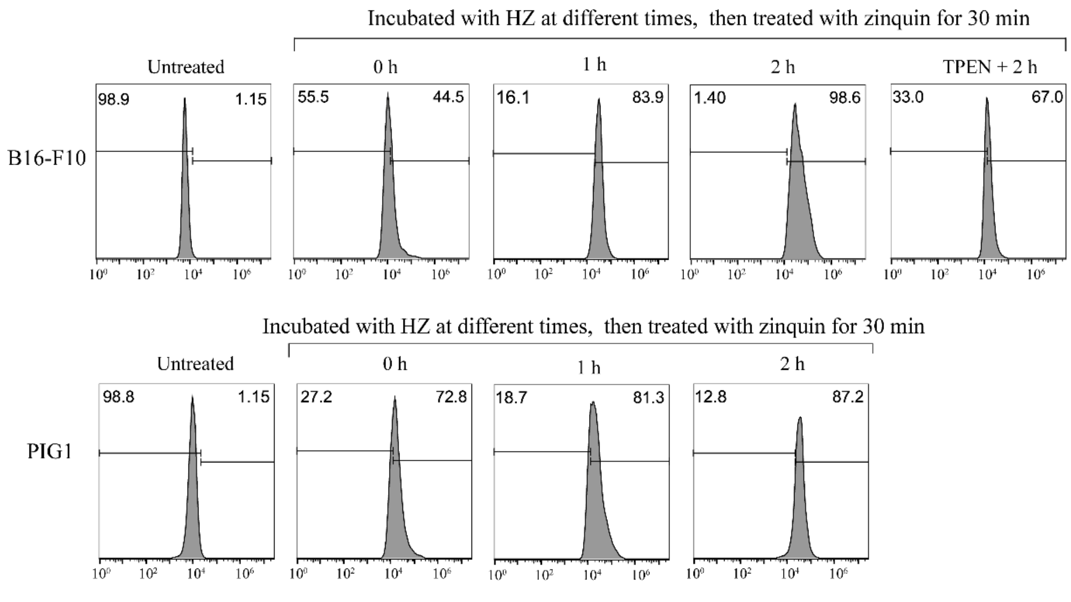

Figure S15. Flow cytometry analysis of differences in Zn^{2+} between B16-F10 and HL-7702 cells.

Analysis of preference of Zn^{2+} accumulation

We analyze the energy exhaustion efficiency of "nano-enabled energy interrupter" on HL-7702 cells (normal hepatocytes). HL-7702 cells were used as control cells, mainly because the liver is the main metabolic organ in the body, and nanoparticles usually have a good liver retention capacity. As shown in Figure S16, although there is little difference in HZ uptake between B16-F10 and HL-7702 cells by adjusting the incubation time (Figure S16a), Zn^{2+} fluctuations in B16-F10 cells are significantly stronger than that in HL-7702 cells (Figure S16b and S16c). Excessive Zn^{2+} accumulation in tumor cells leads to a decrease of NAD^{+} (Figure S16d), which in turn reduces the production of ATP (Figure S16e). For HL-7702 cells, the decrease of intracellular NAD^{+} and ATP was not obvious, which may be due to the insignificant accumulation of intracellular free Zn^{2+}. Therefore, B16-F10 cells can be preferentially killed when treated with HZ of a certain concentration, while cell viability of HL-7702 cells cannot be affected at this concentration (Figure S16f).

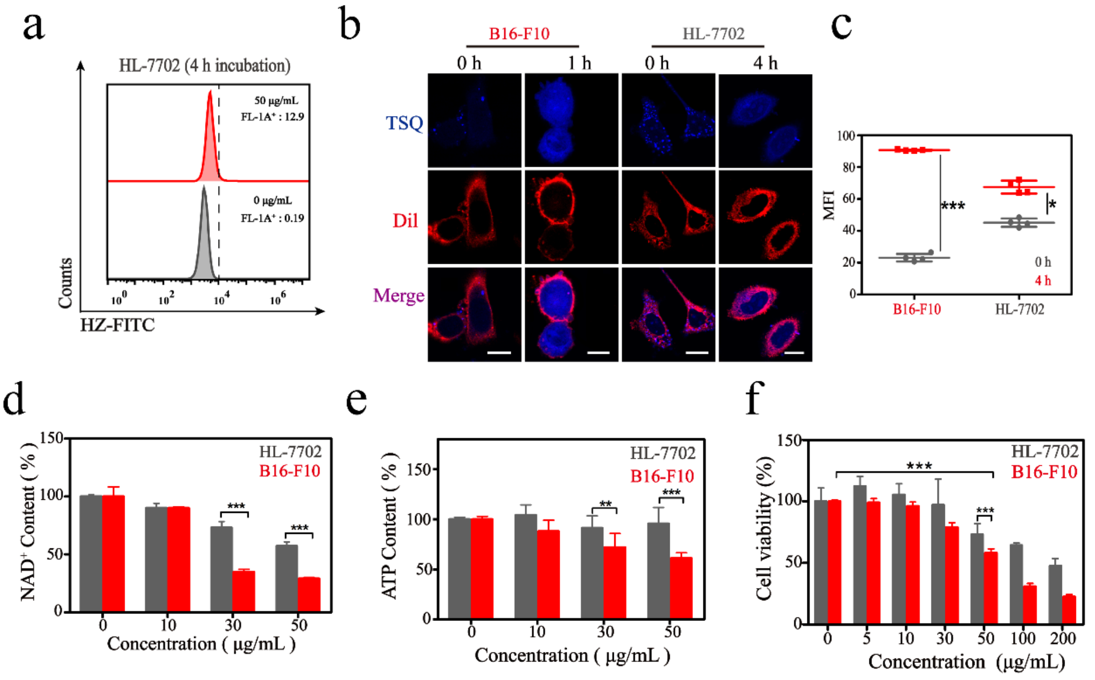

Figure S16. a, Flow cytometry analysis of HL-7702 cells incubated with 50 μg/mL HZ-FITC for 4h. b, c, CLSM images (b), scale bar: 10 μm and Quantitative analysis (c) of intracellular Zn^{2+} production (blue fluorescence) after treatment with HZ on

B16-F10 and HL-7702 cells for 1 h and 4 h, respectively, (n=4). Red fluorescence indicated cell membrane, blue fluorescence indicated free Zn^{2+}. d, e, NAD^+ (d) and ATP (e) levels in B16-F10 or HL-7702 cells treated with different concentrations of HZ (n=3). f, Cell viability of B16-F10 cells and HL-7702 cells subjected to HZ (n=6). All data are shown as the Mean ± SD from three independent experiments. *P*-values were determined by two-tailed unpaired t-test. **P*<0.05, ***P*<0.01, ****P*<0.001, *****P*<0.0001.

Verification of "Zn^{2+} interference"-mediated glycolysis inhibition

To verify the pivotal role of Zn^{2+} overloading induced by HZ in cell death and energy loss, we compared the cell inhibition effect and energy loss ability of HZ, free Zn^{2+} and dimethylimidazole on B16-F10 cells. As expected, HZ NPs and free Zn^{2+} showed markedly obvious inhibition of the viability of B16-F10 cells in a dose-dependent manner, while incubation with equivalent concentrations of dimethylimidazole alone was almost harmless to B16-F10 cells. Meanwhile, TPEN effectively reversed the inhibition effects of free Zn^{2+} on viability of B16-F10 cells. Simultaneously, compared with dimethylimidazole, the content of ATP was obviously inhibited in HZ and free Zn^{2+} concentration-dependent manner in B16-F10 cells, and free Zn^{2+} was ineffective in reducing ATP content in TPEN-pretreated B16-F10 cells. Above all, these results further confirmed the mechanism of "Zn^{2+} interference"-mediated glycolysis inhibition for tumor therapy."

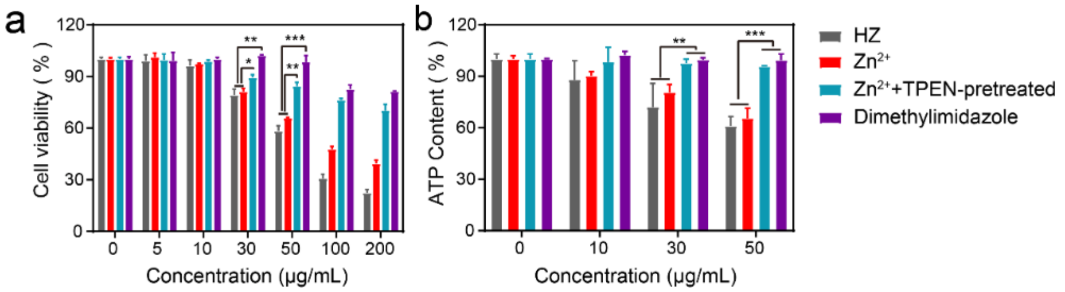

Figure S17. a, b, Cell viability (a) and ATP levels (b) of B16-F10 cells (without or with pre-treated TPEN) subjected to HZ, free Zn^{2+} and dimethylimidazole (n=6). Results are presented as means ± s.d. *P*-values were determined by a two-tailed

unpaired t-test. *$P<0.05$, **$P<0.01$, ***$P<0.001$, ****$P<0.0001$.

KEGG pathway enrichment analysis

KEGG pathway analysis further identified the changes of relevant metabolic pathway induced by HZ treatment on B16-F10 cells. The result revealed that HZ treatment significantly disrupted the metabolic process of B16-F10 cells, including oxidative phosphorylation, purine metabolism, pyrimidine metabolism, glutathione metabolism, fatty acid metabolism and nicotinate and nicotinamide metabolism, et. al.

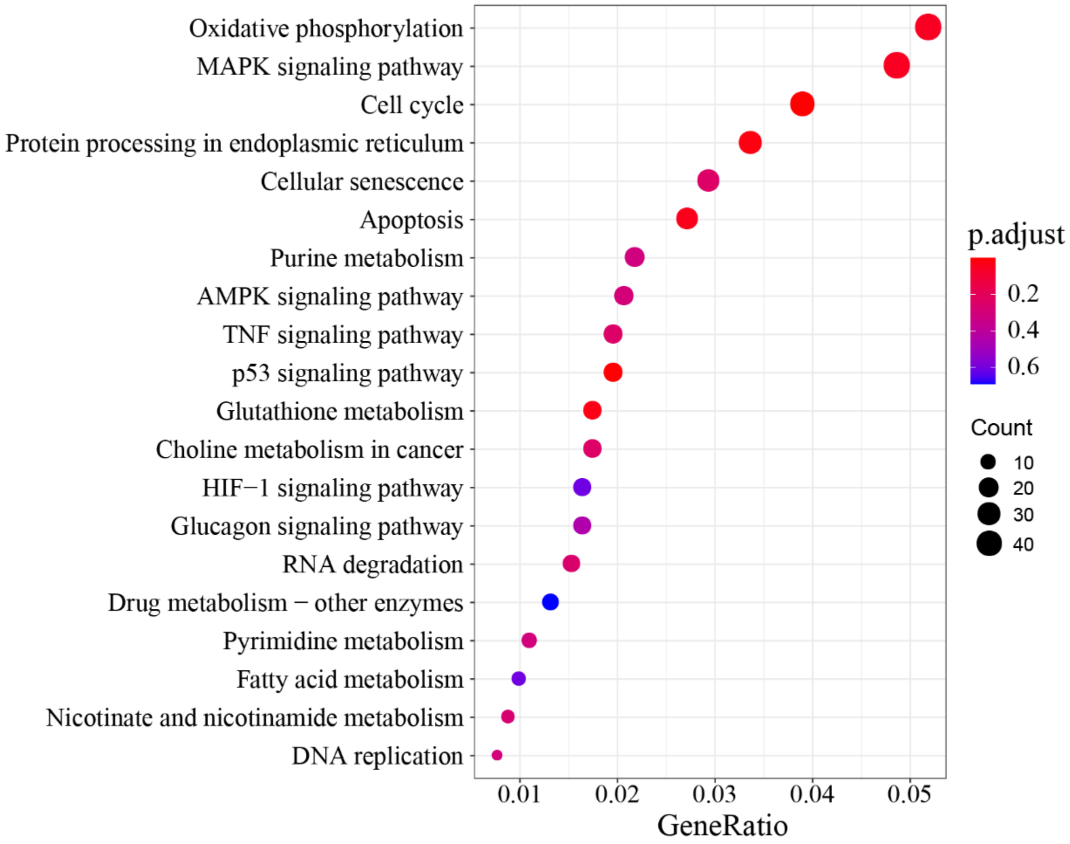

Figure S18. KEGG pathway enrichment analysis of differentially expressed genes between HZ and control treatment group.

Investigation of intracellular uptake efficiency of L@GD-AF488 and HZ@GD-AF488

The difference in cellular uptake level of L@GD-AF488 and HZ@GD-AF488 in B16-F10 cells was investigated by flow cytometer. The result verified that there has a little difference between HZ@GD and L@GD in the cellular uptake efficiency.

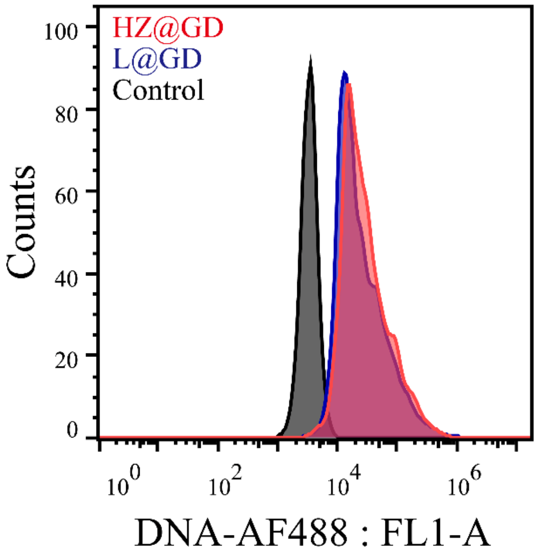

Figure S19. Flow cytometry analysis of B16-F10 cells incubated with L@GD-AF488, HZ@GD-AF488, respectively.

Investigation of the differences of intracellular glucose uptake in B16-F10 cells

The exact uptake level of glucose in B16-F10 cells treated with different formulations was estimated by flow cytometry. B16-F10 cells subjected to HZ@GD exhibited weaker glucose uptake than those control groups, implying that HZ@GD could efficiently restrict glucose into the cells.

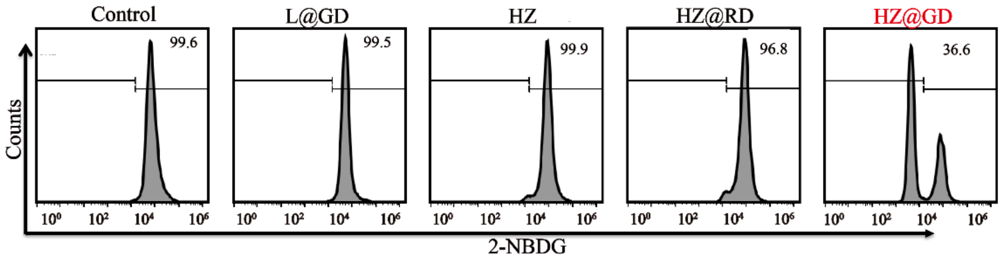

Figure S20. Representative flow cytometry of intracellular glucose level in B16-F10 cells treated with different formulations.

Assay of apoptosis and necrosis of B16-F10 cells

To assess the antitumor potential of HZ@GD, the apoptotic assay based on Annexin V-FITC and propidium iodide staining of B16-F10 cells was conducted. Benefiting from the Zn^{2+}-mediated dual function, HZ@GD triggered a more pronounced apoptosis on B16-F10 cells than other groups. The apoptotic ratio of HZ@GD increased to 91.53%, which was much higher than HZ@RD (49.87%), HZ (51.73%) and L@GD (4.54%).

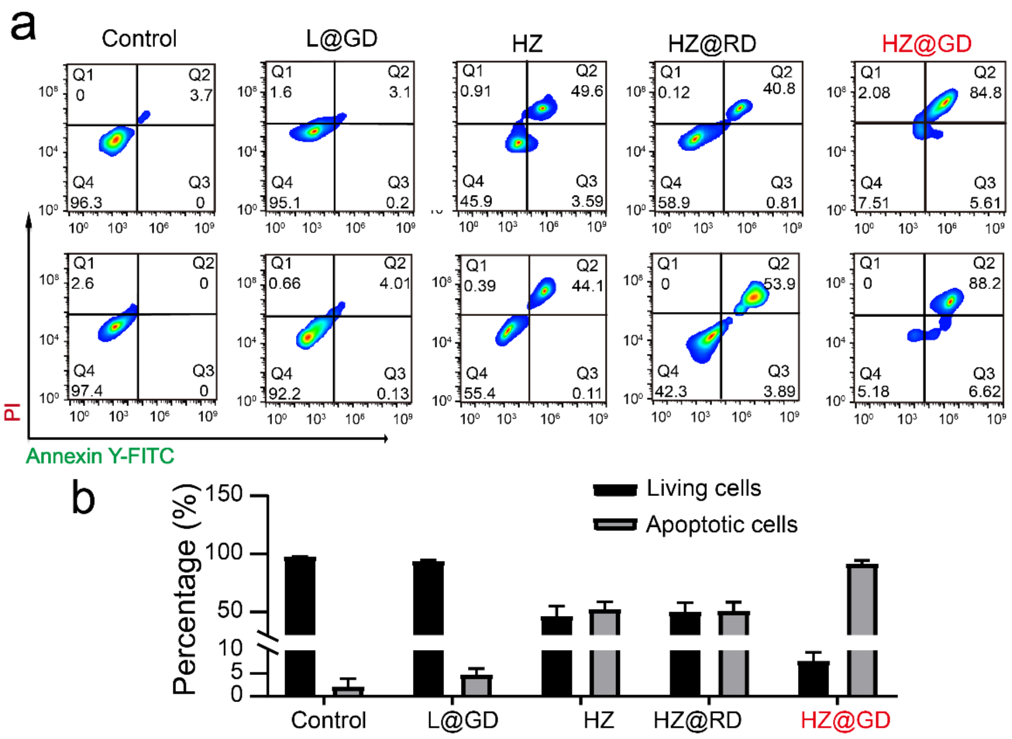

Figure S21. a, b, Flow cytogram (a) and statistical analysis (b, n=3) representing apoptosis and necrosis analysis after treatment with different therapeutic nanoparticles on B16-F10 cells for 24 h. The percentage of living cells is the percentage of Q1, the apoptotic cell rate was calculated by: percentage (%)=Q2+Q3. Results are presented as means ± s.d.

PAGE characterization of cleavage ability of DNAzyme in HL-7702 cells

The cleavage ability of HL-7702 DNAzyme exposure with different concentrations of Zn^{2+} was investigated by PAGE. The catalytic activity increased with increment concentrations of Zn^{2+}. 1 μM of Zn^{2+} was sufficient for conducting a complete substrate cleavage, indicating the comparable cleavage activity between B16-F10 DNAzyme and HL-7702 DNAzyme.

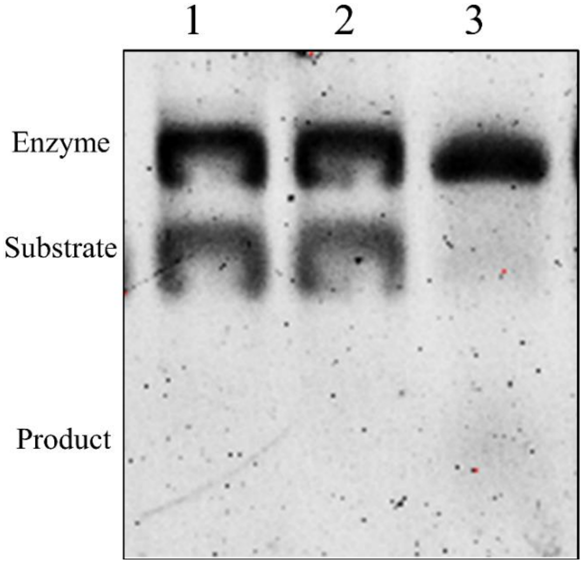

Figure S22. Shearing efficiency of GLUT1 DNAzyme of HL-7702 in the presence of different concentrations of Zn^{2+} by polyacrylamide gel electrophoresis. (1) 100 nM, (2) 500 nM, (3) 1 μM.

Cytotoxicity of HZ@GD on HL-7702 cells

The cytotoxicity of HZ@GD on HL-7702 cells was investigated by CCK8 assay. The cell viability remained 67.9% even with 50 μg/mL of HZ@GD nanoparticles, indicating relatively lower cytotoxicity of HZ@GD on HL-7702 cells compared with B16-F10 cells, which attributed to Zn^{2+}-mediated selective treatment.

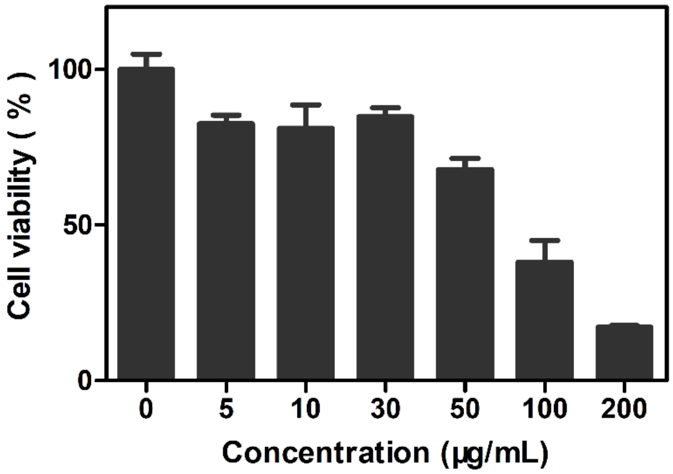

Figure S23. Cell viability of HL-7702 cells incubated with HZ@GD for 24h (n=6). All data are shown as the Mean ± SD from six independent experiments.

Hemolysis assays of HZ@GD

Hemolysis assays were conducted to investigate the biocompatibility of different formulations. No hemolytic reaction was observed with even as high as 1000 μg/mL of HZ@GD nanoparticles, indicating the good compatibility of these nanoparticles.

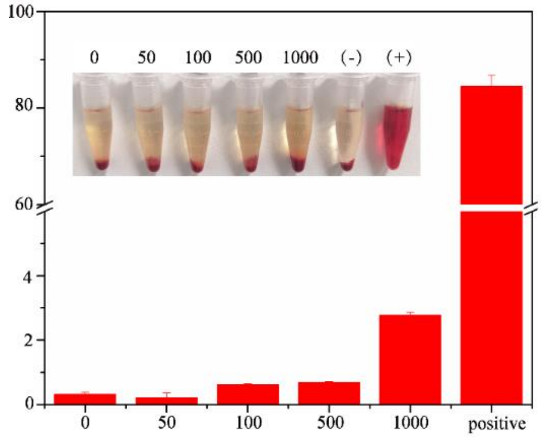

Figure S24. Hemolysis assays of treated with different concentrations of HZ@GD (μg/mL). All data are shown as the Mean ± SD from three independent experiments.

Serum biochemistry analysis of different formulations

Serum biochemistry analysis was also utilized for exploring the potential systematic toxicity of different formulations. All these key parameters of the nanoparticles-treated mice were in good accordance with that of healthy mice, indicating minimal systematic toxicity of the proposed nanoparticles.

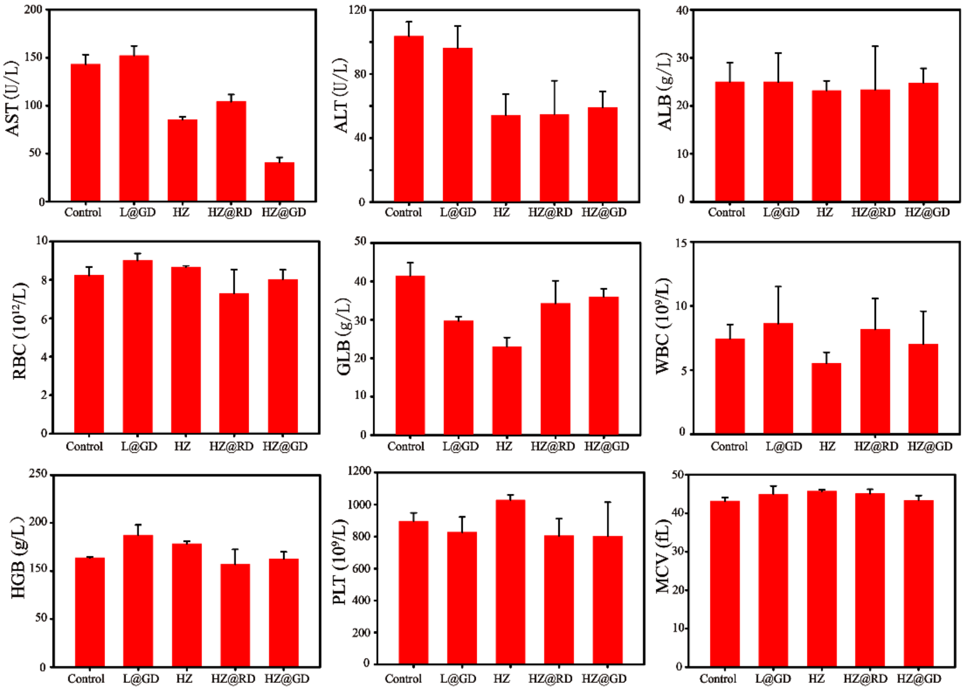

Figure S25. Blood biochemical levels and hematological parameters of the mice after treatment with different groups (n=5). All data are shown as the Mean ± SD from five independent experiments.

In vivo biodistribution of HA/ZIF-8@IR783

A small animal imaging system was utilized for investigating the biodistribution of HA/ZIF-8@IR783 nanoparticles. Profiting from the EPR effect and tumor targeting ability, The HA/ZIF-8@IR783-treated mice showed accumulated fluorescence of IR783 in tumor site, while a relatively lower fluorescence signal was observed in ZIF-8@IR783-treated mice, and there was negligible fluorescence signal was visualized in free IR783-treated mice (Figure S26a-c). These results demonstrated the retention ability of HA/ZIF-8@IR783 in tumor site was stronger than that of free IR783 and ZIF-8@IR783.

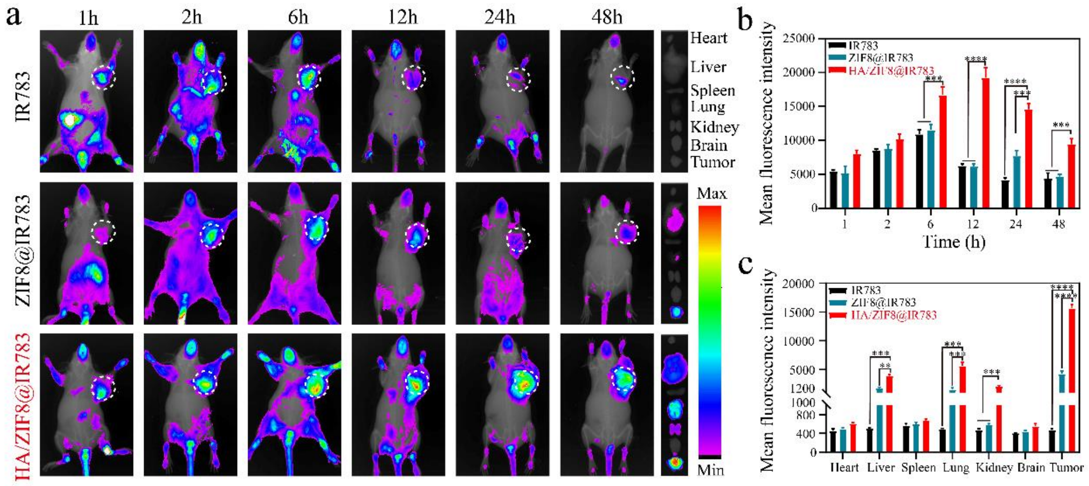

Figure S26. a, Optical living imaging of 4T1 tumor-bearing mice and the major tissues after injection of IR783, Z@IR and HZ@IR for different time-periods and 48 h, respectively. b, Quantitative analysis of NIR FL intensity of 4T1 tumor-bearing mice model after tail vein injected with IR783, Z@IR and HZ@IR for different time-periods, n=3. c, Ex-vivo tissue quantitative analysis of NIR FL intensity of 4T1 tumor-bearing mice model at 48 h post-injection with IR783, Z@IR and HZ@IR, n=3. Results are presented as means ± s.d. P-values were determined by two-tailed unpaired t-test. *P<0.05, **P<0.01, ***P<0.001, ****P<0.0001.

Quantitatively analysis of Zn^{2+} biodistribution of HZ@GD

ICP-MS was carried out to quantitatively analyze Zn^{2+} biodistribution of HZ@GD at 24 h and 48 h postinjection. After treated with HZ@GD, the content of Zn^{2+} increased in the tumor, indicating HZ@GD could effectively accumulate in the tumor site.

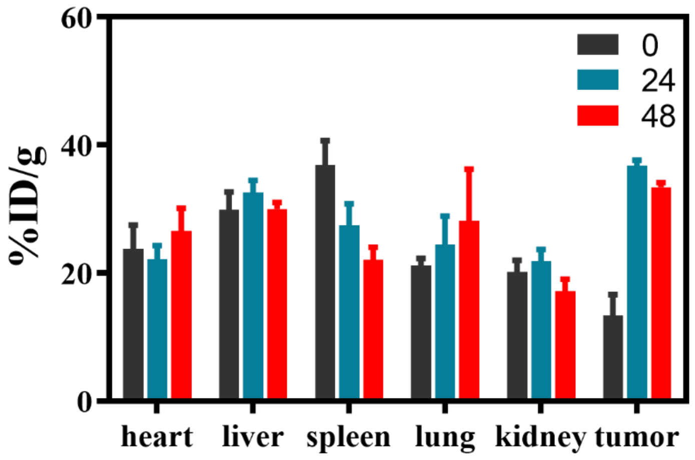

Figure S27. ICP-MS analysis of Zn^{2+} amount in the organs of mice collected at different time points after injection of HZ@GD. ID/g: injected dosage per gram tissues (n=3). All data are shown as the Mean ± SD from three independent experiments.

CLSM images of Zn^{2+} release in tumor site

Zinquin staining was carried out to examine the ability of Zn^{2+} accumulation from HZ@GD in tumor site. Compared with untreated mice, more blue fluorescence of tumor site was observed in HZ@GD-treated mice, indicating the superior ability of "nano-enabled energy interrupter"-induced intracellular Zn^{2+} accumulation.

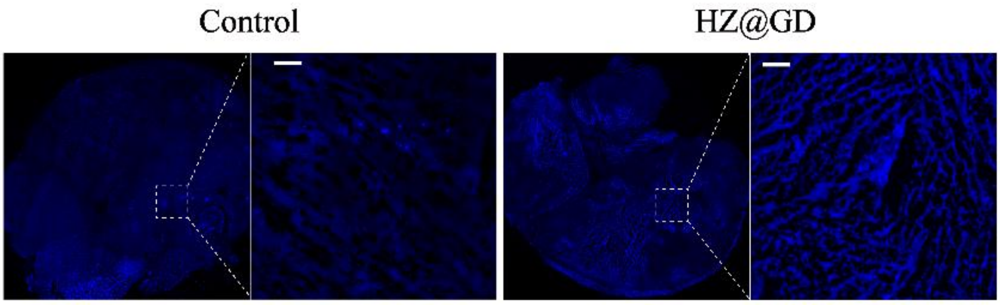

Figure S28. Representative images of Zn^{2+} staining in tumor tissues after treatment with HZ@GD for 24 h. Blue fluorescence indicated free Zn^{2+}. Scale bar: 200 μm.

H&E staining of major organs

To investigate the potential toxicity of different formulations on major organs, the differently treated mice were sacrificed, and the collected heart, liver, spleen, lung, and kidney were analyzed by using hematoxylin and eosin (H&E) staining. No physiological abnormality was visualized in these organs, verifying the minimal systematic toxicity of these nanoparticles.

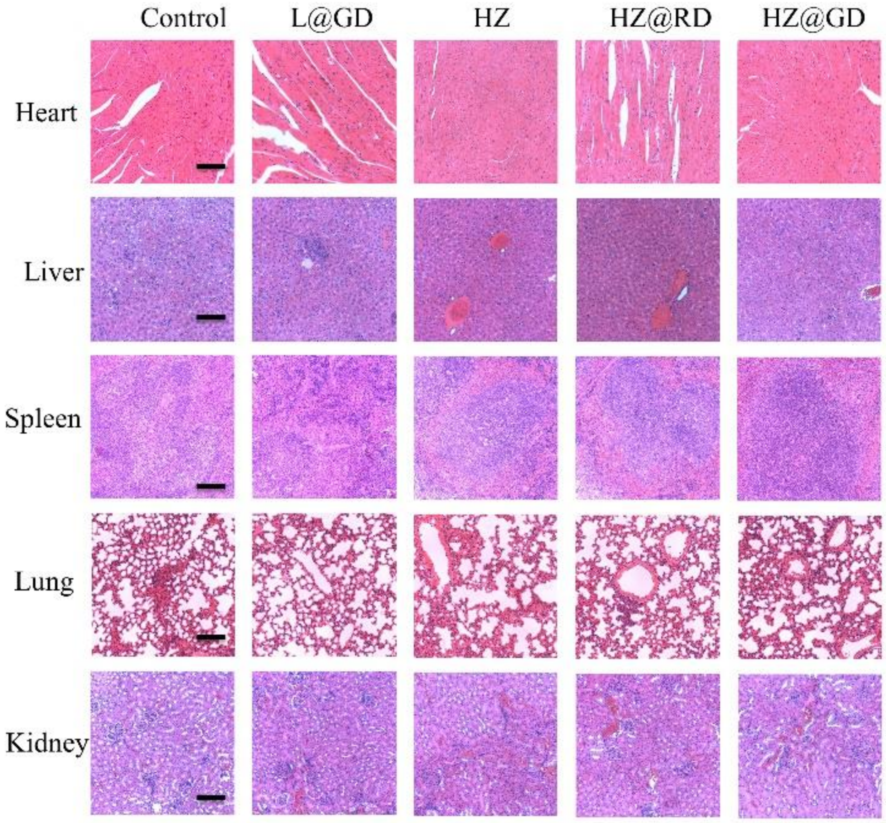

Figure S29. H&E staining of major organs separated from different groups. Scale bar: 200 μm.

Semi-quantitative analysis of fluorescence staining

The semi-quantitative analysis of TUNEL staining and GLUT1 protein staining were conducted for further demonstrating the antitumor effect and mechanism of HZ@GD. As expected, more ration of apoptotic cells in HZ@GD group were observed by semi-quantitative analysis of TUNEL staining (Figure S). To explore the antitumor mechanism of HZ@GD, firstly, the intratumoral GLUT1 expression was analyzed by immunofluorescence assay. As indicated in Figure S, there is little mean fluorescence intensity of red fluorescence (GLUT1) in tumors after treatment of HZ@GD, while high mean fluorescence intensity of red fluorescence could be found in other groups.

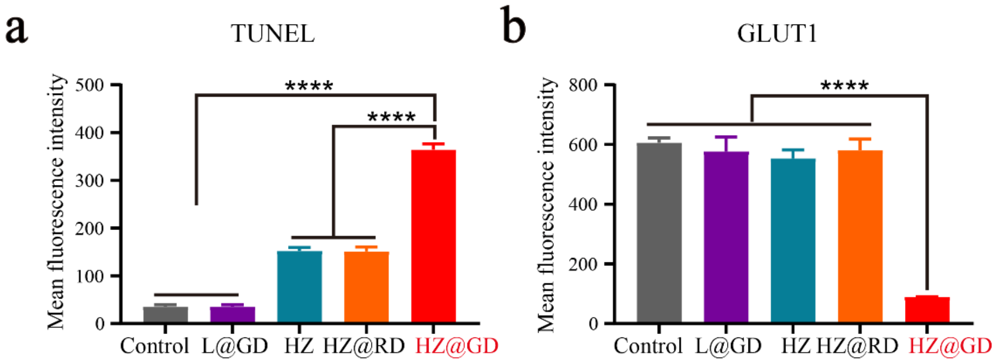

Figure S30. a, b, semi-quantitative analysis of TUNEL staining (a, n=3) and GLUT1 protein immunostaining (b, n=3) in tumor tissues after treatment of various formulations. Results are presented as means ± s.d. *P*-values were determined by two-tailed unpaired t-test. *P<0.05, **P<0.01, ***P<0.001, ****P<0.0001.

Investigation of the expression of intratumoral GLUT1

To investigate the efficiency of Zn^{2+} -mediated gene silencing in vivo, western bolt assay was applied to evaluate the intratumoral GLUT1 expression. The expression of GLUT1 protein was significantly down-regulated in B16-F10 cells incubated with HZ@GD.

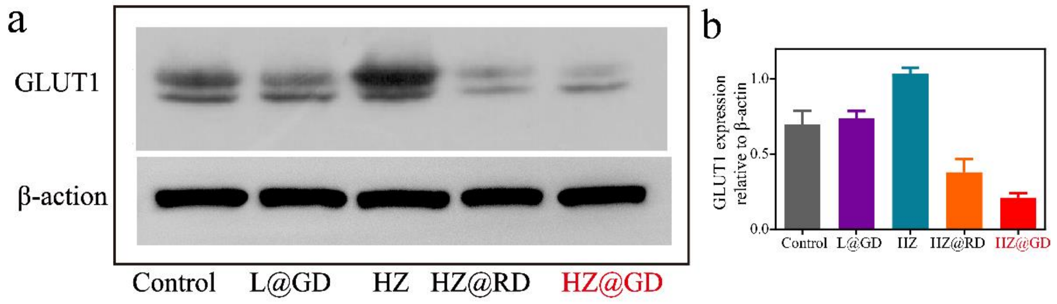

Figure S31. a, b, Western blot (a) and semi-quantitative analysis (b, n=3) of tumor tissue GLUT1 (n=3). All data are shown as the Mean ± SD from three independent experiments.
